# Oxidation inhibits autophagy protein deconjugation from phagosomes to sustain MHC class II restricted antigen presentation

**DOI:** 10.1038/s41467-021-21829-6

**Published:** 2021-03-08

**Authors:** Laure-Anne Ligeon, Maria Pena-Francesch, Liliana Danusia Vanoaica, Nicolás Gonzalo Núñez, Deepti Talwar, Tobias P. Dick, Christian Münz

**Affiliations:** 1 Viral Immunobiology, Institute of Experimental Immunology, University of Zürich, Zürich, Switzerland; 2 Inflammation Research, Institute of Experimental Immunology, University of Zürich, Zürich, Switzerland; 3 Division of Redox Regulation, DKFZ-ZMBH Alliance, German Cancer Research Center (DKFZ), Heidelberg, Germany; 4 Faculty of Biosciences, Heidelberg University, Heidelberg, Germany

**Keywords:** Macroautophagy, Phagocytosis, MHC class II

## Abstract

LC3-associated phagocytosis (LAP) contributes to a wide range of cellular processes and notably to immunity. The stabilization of phagosomes by the macroautophagy machinery in human macrophages can maintain antigen presentation on MHC class II molecules. However, the molecular mechanisms involved in the formation and maturation of the resulting LAPosomes are not completely understood. Here, we show that reactive oxygen species (ROS) produced by NADPH oxidase 2 (NOX2) stabilize LAPosomes by inhibiting LC3 deconjugation from the LAPosome cytosolic surface. NOX2 residing in the LAPosome membrane generates ROS to cause oxidative inactivation of the protease ATG4B, which otherwise releases LC3B from LAPosomes. An oxidation-insensitive ATG4B mutant compromises LAP and thereby impedes sustained MHC class II presentation of exogenous *Candida albicans* antigens. Redox regulation of ATG4B is thereby an important mechanism for maintaining LC3 decoration of LAPosomes to support antigen processing for MHC class II presentation.

## Abstract (web summary)

LC3-associated phagocytosis (LAP) is a non-canonical use of the autophagy machinery that can contribute to immune responses. Here, the authors describe the mechanism by which ROS production regulates LAPosome stabilization sustaining MHC II dependent antigen presentation.

## Introduction

Macroautophagy (hereafter autophagy) contributes to cell intrinsic, innate, and adaptive immunity^1,2^. It has been shown to restrict intracellular pathogens, regulate innate inflammatory responses such as inflammasome activation, influence lymphocyte metabolism, as well as to contribute to antigen presentation to T cells^1,2^. Our group and others have demonstrated the involvement of the autophagy machinery during adaptive immunity by inhibiting or promoting antigen presentation on major histocompatibility complex (MHC) class I and II molecules, respectively^3–10^. The molecular mechanisms involved during autophagy, which requires the highly regulated activity of autophagy-related proteins (ATGs), to form a double membrane vesicle, named autophagosome, and for its delivery to lysosomal degradation are now well understood^11^. So far, three major protein complexes are involved in autophagosome generation. Most upstream is the ULK1/2 complex, and upon starvation, its kinase activity is required for the activation of the class III PI3-kinase (PI3K) complex, which includes the core proteins VPS15, VPS34, and Beclin-1. The activated kinase VPS34 is responsible for the production of phosphatidylinositol 3-phosphate (PI(3)P) marks at the site of autophagosome formation, often at the endoplasmic reticulum, allowing the recruitment of the downstream autophagy proteins. Several ATG proteins, such as WIPIs and ATG16L1, help the recruitment of the last complex in autophagosome generation: the ATG8/LC3 conjugation complex, consisting of ATG5, ATG12, and ATG16L1. This complex conjugates in a ubiquitin-like reaction LC3A/B/C, GABARAP, and GABARAPL1/2 (yeast ATG8 orthologues) not to proteins but to the lipid phosphatidylethanolamine present in the membrane of the forming autophagosome, which in turn mediates cargo recruitment and autophagosome elongation at this site. Thereby, the membrane labeling with LC3 proteins results in de novo autophagosome formation. This membrane conjugation of LC3 family members is dependent on the cysteine protease ATG4. Four orthologues of yeast ATG4 exist in mammalian cells (ATG4A-D) and process pro-LC3 proteins by liberating a C-terminal glycine 120 (LC3-I form), allowing its lipidation (LC3-II form) to the autophagosomal membrane. In addition to processing LC3 precursors, ATG4 plays another crucial role in the deconjugation of these proteins. ATG4 cleaves lipidated LC3 at the glycine 120 from the outer autophagosomal membrane during vesicle maturation, in order to release and recycle cytosolic LC3^12^.

Interestingly, a noncanonical form of autophagy, known as LC3-associated phagocytosis (LAP), has also been described to be involved in a wide range of cellular processes, including immune regulation and inflammatory responses^13,14^. LAP is independent of the ULK1/2 complex but seems to require the ATG8/LC3 conjugation complex and is characterized by LC3B attachment to the cytosolic side of the phagosome membrane^15^. This leads to the formation of a single-membrane vesicle decorated by LC3, named the LAPosome. LAP is activated by the engagement and subsequent engulfment of cell surface receptors, including Toll-like receptor 2 (TLR2), dectin-1, FcR, or TIM-4^14,16–19^. These receptors recognize a variety of ligands such as dying cells, immune complexes, and pathogen moieties, triggering the activation of a subset of ATG proteins, which allows the conjugation of LC3B to the phagosome to ultimately fuse with the lysosome. Although LAP occurs in a wide range of tissues and cell types, the outcome of this pathway is cell type dependent. It appears that in mouse myeloid cells (macrophages), LAP promotes the fusion of LAPosomes with lysosomes to accelerate pathogen clearance, whereas in mouse plasmacytoid dendritic cells, LAP redirects endocytosed cargo to TLR9 containing endosomes^14–16^. However, in human macrophages and conventional dendritic cells, LAP stabilizes and maintains antigens, resulting in prolonged MHC class II presentation^20^. LAP shares various key regulators with autophagy, but also differs in its molecular machinery. Among the differences, the production of reactive oxygen species (ROS) by NADPH oxidase 2 (NOX2) is exclusively required for LAPosome formation^15,20^. NOX2 is responsible for ROS production and pH regulation during phagosome maturation in macrophages, in order to successfully control infections^21^. How ROS generation and specifically how NOX2 promotes LAPosome formation remains unclear. Therefore, we aimed to clarify by which mechanism NOX2 supports LAP. It has recently been shown that the WD40 domain of ATG16L1 supports the lipidation of LC3 and thus its attachment to the phagosomal outer membrane in a NOX2 independent fashion^15,22^.

In this work, we demonstrate that LAPosomes blocked in their maturation are decorated with NOX2 and present high and sustained ROS production. We also show that the LC3-delipidation activity of the protease ATG4B is redox regulated, i.e., that oxidation of a critical cysteine residue close to the active site leads to inactivation of ATG4B-delipidation activity and abolishes LAPosome maintenance. These findings suggest that the inhibition of LC3B delipidation from the membrane by NOX2-derived ROS production stabilizes LAPosomes and favors a prolonged MHC class II presentation of LAPosome cargo. Together with a study that demonstrates ATG4 inhibition by ULK1 during autophagosome formation^23^, this work suggests that ATG4B contributes to the membrane specificity of LC3 protein lipidation both during autophagy and LAP.

## Results

### Complexes containing VPS34/ATG14 or ATG16L1 are recruited to LAPosomes

**Fig. 1:**
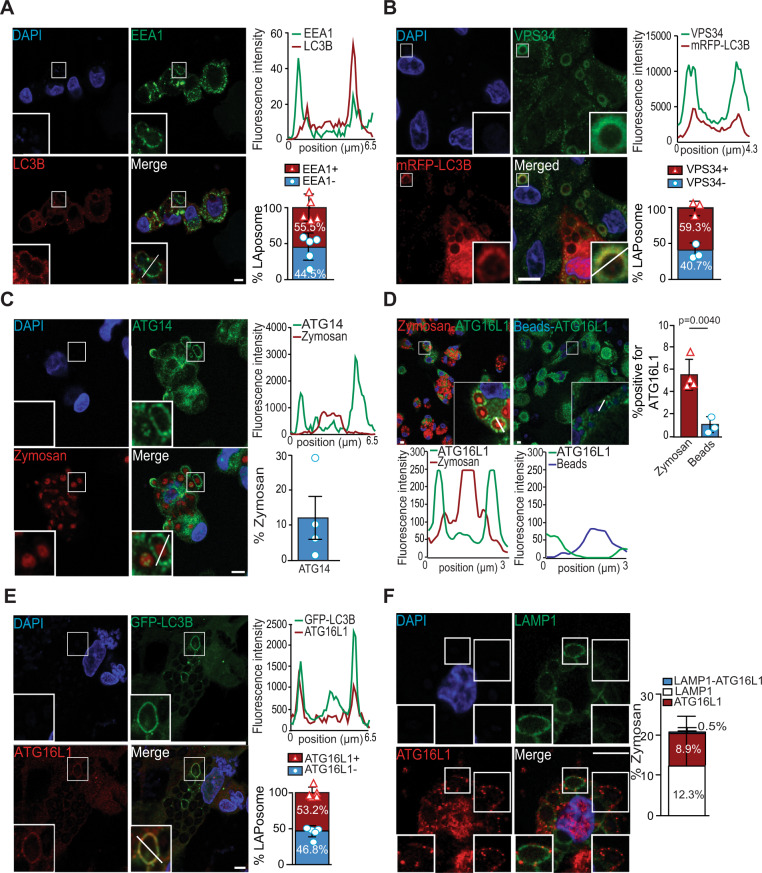
The VPS34/ATG14 and ATG16L1 complexes are recruited to LAPosomes. **A** Human macrophages stimulated with zymosan for 1 h and costained for EEA1 (green) and LC3B (red). Bar graph shows the percentage of LAPosomes colocalizing with EEA1; *n* = 5; each symbol represents an individual experiment. **B** mRFP-LC3B transduced macrophages stimulated with zymosan for 6 h and stained for VPS34 (green). Bar graph shows the percentage of LAPosomes in mRFP/GST-LC3B cells colocalizing with VPS34. Bar represents mean ± SD of data pooled from three independent experiments and each symbol represents an individual experiment. **C** Macrophages stimulated with zymosan-Texas Red for 1 h and stained for ATG14 (green). Graph shows the percentage of zymosan-containing phagosomes displaying ATG14, each symbol represents an individual experiment. Bar represents the mean ± SD of three independent experiments quantified by two independent investigators. **D** Macrophages stimulated with zymosan-Texas Red (red) or with inert beads (blue) for 6 h and stained for ATG16L1 (green). Quantification of the percentage of zymosan or beads positive for ATG16L1 is shown in the bar graph. Bars represent the mean ± SD; data pooled from *n* = 4 (zymosan) and *n* = 3 (beads) independent experiments, quantified by two independent investigators and each symbol represents an individual experiment; unpaired Student’s *t* test, two-tailed. **E** GFP-LC3B transduced macrophages stimulated with zymosan for 6 h and stained for ATG16L1 (red). Bar graph shows the quantification of the percentage of LAPosomes in GFP/GST-LC3B cells colocalizing with ATG16L1; *n* = 5 and each symbol represents an individual experiment. **A**–**E** Fluorescence intensities were quantified along the white segment as shown in the merged panel and plotted as a histogram. **F** Macrophages stimulated with zymosan for 6 h and costained for LAMP1 (green) and ATG16L1 (red). Left-bottom inserts show a zymosan-containing phagosome decorated with LAMP1 but not ATG16L1. Right-bottom inserts show a zymosan-containing phagosome negative for LAMP1 but positive for ATG16L1. Bar graph shows the percentage of zymosan-containing phagosomes displaying single staining for LAMP1 or ATG16L1, or both. The bar graph represents the mean ± SD of three independent experiments, quantified by two investigators blinded to the experimental conditions. All confocal images are representative images of three different independent experiments. Scale bar is 5 µm, and inserts are zoomed 5× from white-framed regions of the immune fluorescence micrographs. Source data are provided as a Source Data file.

Regulation of autophagy requires the sequential involvement of several protein complexes starting with the ULK1/2 complex, followed by the class III PI3K complex and then by the LC3 conjugation complex^11^. However, the molecular mechanisms involved in LAP regulation remain to be further elucidated. There is evidence from murine macrophages that LAP regulation is ULK1/2-independent but requires the involvement of the PI3K complex and the LC3 conjugation complex to successfully form single-membrane LC3 positive vesicles, termed LAPosomes^15^. By analogy with phagocytosis, where it has been reported that the early endosome marker Rab5 directly interacts with VPS34 to sustain phagosome maturation^24,25^, we examined whether early endosome markers were associated with LAPosomes. Human monocyte-derived macrophages were stimulated with the well-known LAP-trigger zymosan for 1 h^14^. We first observed that zymosan-containing phagosomes were surrounded by the autophagic protein LC3B, confirming LAPosome formation and that the majority were also decorated with EEA1 (Fig. 1A). The histogram of the phagosome cross-section revealed an increase in fluorescent intensities associated with EEA1 or LC3B on either side of the zymosan particle (Fig. 1A). We also observed that a small portion of EEA1-decorated zymosan vesicles was also positive for VPS34 (Supplementary Fig. 1a). Furthermore, the recruitment of the class III PI3K complex formed by VPS34 and ATG14 to LAPosomes was examined. After 6 h of stimulation with zymosan, almost 60% of LAPosomes were also positive for VPS34 (Fig. 1B and Supplementary Fig. 1b). Inhibition of VPS34 with the SAR405 inhibitor^26^ decreased the ability of macrophages to form LAPosomes, confirming the involvement of VPS34 in LAPosome formation (Supplementary Fig. 1c). VPS34 is the core protein of several class III PI(3) kinase complexes and is associated with VPS15, Beclin-1, Ambra1, and ATG14 for autophagosome formation. To investigate if this class III PI3K complex is recruited to LAPosomes, we investigated the presence of ATG14 upon zymosan stimulation in human macrophages and observed that a small fraction of zymosan-containing phagosomes displayed ATG14 (Fig. 1C). Moreover, the results suggest that the VPS34/Beclin-1/ATG14 complex is recruited to early LAPosomes and at the same time as the early endosome protein EEA1/Rab5. VPS34 generates PI(3)P, which serves as docking site to recruit the LC3 conjugation complex containing ATG12, ATG5, and ATG16L1 to form autophagosomes. Therefore, the recruitment of the LC3 conjugation complex during LAP was investigated. After 6 h of stimulation, we observed that two members of the LC3 conjugation complex: ATG16L1 and ATG12 were preferentially recruited to zymosan-containing phagosomes compared to the negative control, inert beads (Fig. 1D and Supplementary Fig. 2a, b). ATG16L1 was found four times more frequently associated with zymosan than with inert beads-containing phagosomes (Fig. 1D). To assess whether these zymosan-containing phagosomes are LAPosomes, we investigate the colocalization of ATG16L1 or ATG12 with LC3B. After 6 h of LAP stimulation, we observed colocalization of LC3B-decorated zymosan-containing phagosomes with ATG16L1 (Fig. 1E). Quantitative analysis showed that 53.2% of LAPosomes were positive for ATG16L1 (Fig. 1E and Supplementary Fig. 2d). Similar results were observed for ATG12 (Supplementary Fig. 2a–c). In line with our previous findings, these results showed that the ATG12–ATG16L1–ATG5 complex is recruited and required for the LAP pathway^20^. Altogether, these results suggest that the LC3 conjugation complex colocalizes with the VPS34/ATG14 complex upon LAP stimulation.

### LAPosomes display NOX2 and an elevated oxidation required for LAP

**Fig. 2:**
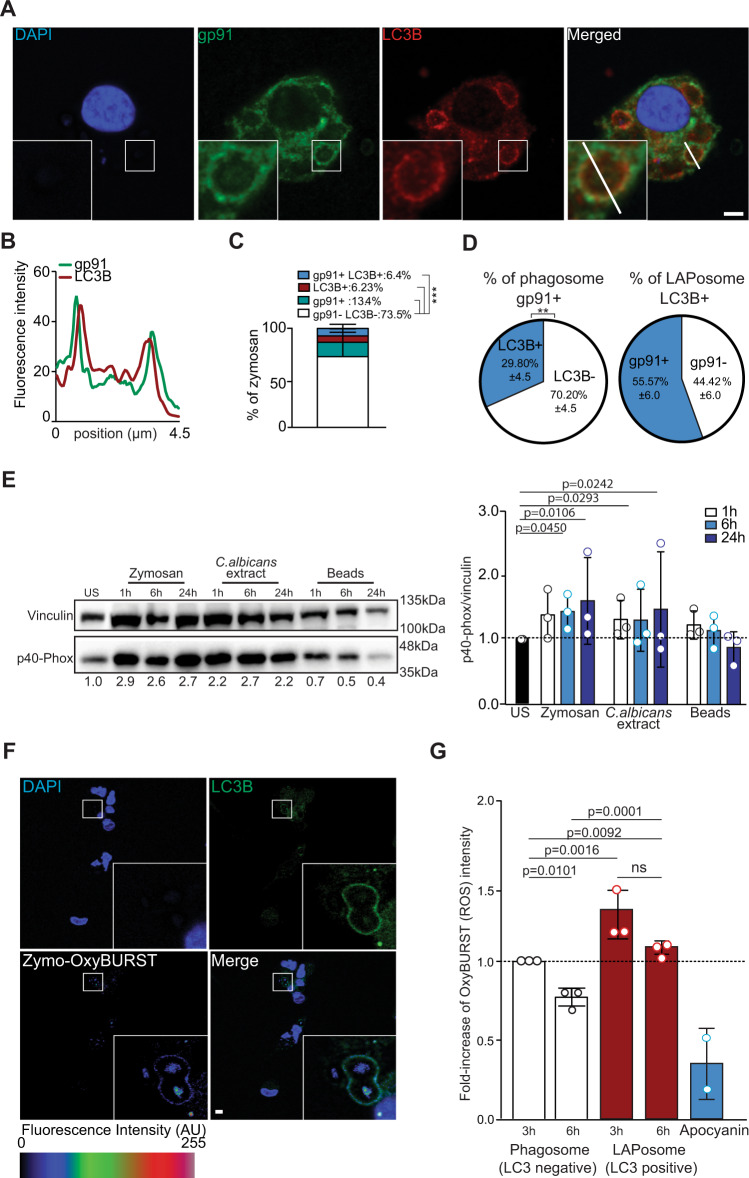
Elevated ROS levels inside LAPosomes are maintained for several hours. **A** Human macrophages were stimulated with zymosan for 6 h, fixed and costained for gp91 (green) and LC3B (red). **B** Histogram shows the fluorescence intensity of gp91 (green) and LC3 (red) along the white segment in the merged fluorescence panel of **A**. Confocal images are representative of three different independent experiments. Scale bar is 5 µm, and inserts are zoomed 5× from white-framed regions of the immune fluorescence micrographs. **C** Quantitative analysis of the percentage of zymosan-containing vesicles displaying only LC3 or gp91, both or none. Bar graph represents the mean ± SD of three independent experiments, quantified by two independent investigators. One-way ANOVA test: ***p < 0.005. **D** The first pie chart shows the percentage of gp91^+^ phagosomes displaying LC3B. Second pie chart on the left shows the percentage of LAPosome (LC3B^+^) colocalizing with gp91, Mann–Whitney test, two-tailed: **p < 0.005. **E** Macrophages were stimulated with zymosan, *Candida albicans* extract, or beads for 1 up to 24 h, lysed and the change on p40-phox protein level were assessed by western blotting. One representative experiment of three is shown. Bars represent the mean of band intensity of p40-phox normalized to the loading control vinculin from three independent experiments (symbols represent one experiment). All conditions are normalized to the unstimulated condition (US), which is set to 1. One-way ANOVA test. **F** Macrophages were stimulated with OxyBURST-coated zymosan for 6 h, fixed and stained for LC3 (red). OxyBURST intensity levels were measured inside LAPosomes and LC3-negative phagosomes. Confocal images are representative of three different independent experiments. Scale bar is 5 µm, and inserts are zoomed 5× from white-framed regions of the immune fluorescence micrographs. **G** Bar graph represents OxyBURST intensity increase after 3 or 6 h of OxyBURST-coated zymosan stimulation inside phagosomes or LAPosomes. All conditions are normalized to the condition of phagosome after 3 h of stimulation, which is set to 1. Bars represent the mean ± SD of three independent experiments (symbols represent one experiment) and for each, more than 50 LAPosomes and phagosomes were analyzed. One-way ANOVA test. Source data are provided as a Source Data file.

**Fig. 3:**
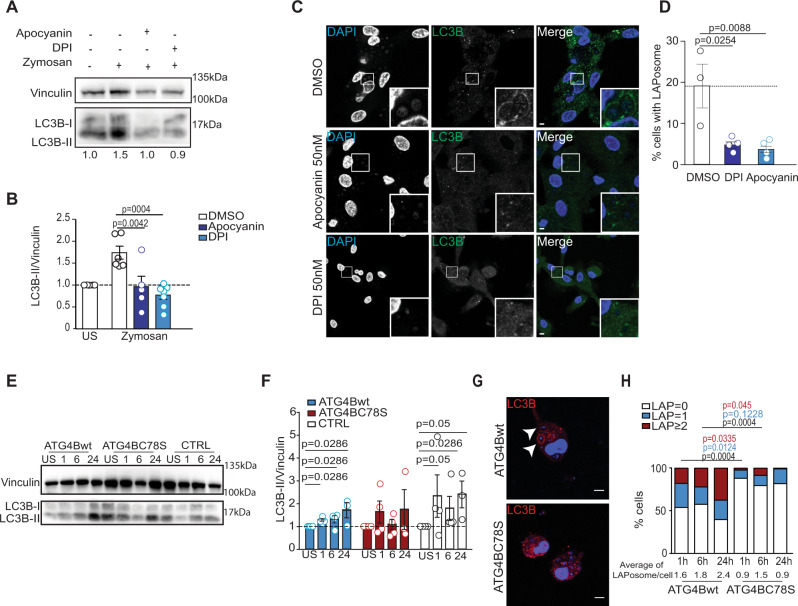
Regulation of ATG4B-delipidation activity by ROS is involved in LAPosome stabilization. **A** Macrophages pretreated with NOX inhibitors were stimulated with zymosan for 6 h and then lysed. Cell lysates were subjected to SDS-PAGE gel electrophoresis and immunoblotted for LC3B and the loading control vinculin. One representative experiment of three is shown and US represents unstimulated conditions. **B** Bar graph shows the level of LC3B-II protein expression normalized to vinculin. All conditions are normalized to the DMSO unstimulated condition, which is set to 1. Bars represent the mean ± SD: DMSO (*n* = 6), apocynin (*n* = 5), and DPI (*n* = 7) independent experiments, and each symbol represents a single experiment, ordinary one-way ANOVA. **C** Macrophages were pretreated with NOX inhibitors (apocynin 50 nM and DPI 50 nM) and stimulated for 6 h with zymosan, fixed and stained for LC3B (green). Representative images from three independent experiments are shown, scale bar is 5 µm, and inserts are 5× zoomed from white-framed regions in the original images. **D** Bar graph displays the percentage of cells with LAPosomes in each condition. Mean of three independent experiments and each symbol represents a single experiment. Unpaired *t* test two-tailed. **E**, **F** Macrophages transduced with lentiviruses encoding Flag, Flag-ATG4Bwt, or Flag-ATG4BC78S were stimulated with zymosan for 1–24 h, lysed, and LC3B protein levels were assessed by western blot. Image from one representative of three independent experiments. Bar graph shows the level of LC3B-II expression normalized to vinculin. All conditions are normalized to the respective unstimulated conditions (US), which are set to 1, and bars represent mean ± SD of three independent experiments, and each symbol represents a single experiment. Mann–Whitney test, two-tailed. **G** Macrophages transduced with lentiviruses encoding Flag-ATG4Bwt or Flag-ATG4BC78S were stimulated with zymosan for 6 h, fixed and stained for LC3B (red). White arrows indicate the recruitment of LC3B to zymosan-containing phagosomes. Representative images from three independent experiments are shown with scale bar of 5 µM. **H** Bar graph shows the percentage of LAPosomes formed per cell upon zymosan stimulation (1 h up to 24 h) in macrophages overexpressing Flag-ATG4Bwt or Flag-ATG4BC78S. Bars represent the fraction of total mean of five independent experiments. One-way ANOVA test. Source data are provided as a Source Data file.

In this study, we confirmed that LAPosomes seem to have a delay in maturation in human macrophages. Indeed, ATG16L1-decorated zymosan-containing phagosomes were rarely positive for the lysosomal marker LAMP1. After 6 h of zymosan stimulation, only 0.5% were decorated with both ATG16L1 and LAMP1, while 12.3% and 8.9% were decorated with only ATG16L1 or LAMP1, respectively (Fig. 1F). Furthermore, the percentage of LAMP1-positive zymosan-phagosomes decreased over time to undetectable levels at 24 h after stimulation, while ATG16L1-positive and zymosan-containing phagosomes were still observed at this time point (Supplementary Fig. 2e). Therefore, we addressed the mechanism of LAPosome stabilization. Our group had previously demonstrated that macrophages produced high levels of ROS after LAP stimulation and that LC3B accumulates on phagosomal membranes in a NOX2-dependent manner^20^. NOX2 is a protein complex formed by transmembrane and cytosolic proteins, which is only active when all the subunits come together at the phagosomal membrane^27^. We wondered if the delay of LAPosome maturation could be orchestrated by the recruitment of NOX2 and tested whether NOX2 subunits can be found associated with LAPosomes. The transmembrane subunit gp91 was found associated with LC3B positive and zymosan-containing phagosomes (Fig. 2A–C). Overall, 30% of gp91 positive phagosomes contained LC3B, whereas 55% of LAPosomes colocalized with gp91, indicating that NOX2 is preferentially recruited to LAPosomes (Fig. 2D). The recruitment of a second transmembrane subunit of NOX2, p22-phox, was also observed and p22-phox was detected at zymosan-containing phagosomes up to 24 h after LAP stimulation (Supplementary Fig. 3a). Interestingly, the levels of the cytosolic subunit p40-phox were significantly increased upon LAP stimulation and p40-phox was recruited to zymosan-containing phagosomes, indicating that the active NOX2 complex is present at LAPosomes (Fig. 2E and Supplementary Fig. 3b). In view of NOX2 recruitment to LAPosomes, its ability to produce ROS toward the inside of LAPosomes was investigated. To address this point, we used the fluorescent probe OxyBURST, whose fluorescence intensity increases with oxidation. Macrophages were stimulated with OxyBURST-coupled zymosan, and we confirmed that OxyBURST fluorescence intensity could be increased by hydrogen peroxide (H_2_O_2_) exposure and this was not affected by coupling to zymosan (Supplementary Fig. 3c). Human macrophages stimulated with *Candida albicans* extract or OxyBURST-coated zymosan showed a significant increase in LC3B-II protein levels, compared to the unstimulated condition (Supplementary Fig. 3d, e). Furthermore, OxyBURST-coated zymosan was also observed in LAPosomes, indicating that OxyBURST coupling did not affect the ability of zymosan to trigger LAP (Fig. 2F and Supplementary Fig. 3f). The levels of oxidation measured within LAPosomes were significantly higher than the level detected inside LC3-negative phagosomes (Fig. 2F, G). This observation was further confirmed by measuring the OxyBURST fluorescence intensity from z-stack image projections (Supplementary Fig. 3f, g). Interestingly, the oxidation levels in phagosomes were significantly reduced overtime, while they remained elevated and stable within LAPosomes (Fig. 2F, G). To go one step further in the understanding of the role of NOX2 in LAPosome formation, we chemically inhibited ROS by pretreating macrophages with two NOX inhibitors: apocynin or diphenyleneiodonium chloride (DPI), for 1 h before LAP stimulation. Macrophages pretreated with NOX inhibitors displayed less LC3B-II accumulation upon zymosan stimulation, compared to untreated conditions and NOX inhibitors did not affect the basal LC3B-II levels measured in unstimulated and inert-bead stimulated conditions (Fig. 3A, B and Supplementary Fig. 3h, i). The ability of macrophages to form LAPosomes was fourfold reduced by NOX inhibition (Fig. 3C, D). In addition, treatment with NOX inhibitors was associated with a decreased oxidation level within phagosomes (Fig. 2G). Together with our previous work, these results demonstrate that NOX2 is required for LAPosome formation and maintenance^20^. Its activity seems to sustain elevated oxidation within LAPosomes.

### ATG4B-delipidation activity is inhibited by ROS during LAP

ROS have been reported to regulate autophagy^28,29^. Subsequent studies have shown that ROS are essential for starvation induced autophagy by regulating key ATGs proteins, such as ATG3, ATG7, or ATG4^30–32^. Scherz-Shouval et al. recently reported that autophagosome formation is blocked by antioxidants and that cysteine proteases ATG4A and ATG4B are sensitive to oxidation by H_2_O_2_. They identified cysteine residue Cys-81 in ATG4A, corresponding to Cys-78 in ATG4B, as essential for oxidative inhibition of LC3 delipidation as catalyzed by these proteases^30^. Based on our findings, we examined whether NOX2-generated ROS regulate ATG4B-delipidation activity during LAP. To address this question we used the ATG4BC78S mutant, previously shown to be oxidation insensitive^30^. The osteosarcoma cell line U2OS, either overexpressing Flag-ATG4Bwt or Flag-ATG4BC78S, was used to confirm that Cys-78 of ATG4B facilitates redox regulation of its delipidation activity. The level of LC3B lipidation in U2OS cells overexpressing Flag-ATG4Bwt was increased upon H_2_O_2_ exposure with drugs stimulating macroautophagy (rapamycin) or inhibiting the autophagosome degradation (bafilomycin A1) (Supplementary Fig. 4a). Accumulation of lipidated LC3B was not observed in cells overexpressing Flag-ATG4BC78S, suggesting that this mutant is insensitive to oxidative inactivation (Supplementary Fig. 4a). To investigate whether redox regulation of ATG4B plays a role during LAP, human macrophages were transduced with lentiviruses encoding Flag-ATG4Bwt or Flag-ATG4BC78S, and stimulated with zymosan (Fig. 3E–H and Supplementary Fig. 4b). No significant differences of expression between the oxidation-insensitive mutant and ATG4Bwt were observed. However, LC3B-II protein levels were significantly elevated upon zymosan stimulation in controls and Flag-ATG4Bwt transduced macrophages, but not upon Flag-ATG4C78S expression (Fig. 3E, F and Supplementary Fig. 4b). Furthermore, the expression of Flag-ATG4Bwt in macrophages did not affect the recruitment of LC3B to zymosan-containing phagosomes, whereas LAPosome accumulation was compromised upon Flag-ATG4BC78S expression (Fig. 3G). The majority of Flag-ATG4Bwt expressing macrophages were able to form one or more LAPosomes upon zymosan stimulation, while only up to 20% of macrophages overexpressing Flag-ATG4BC78S were able to form mostly only one LAPosome, suggesting that delipidation by the oxidation-insensitive ATG4B mutant compromises LAP (Fig. 3H). Expression of the Flag tag alone in macrophages did not inhibit LAPosome formation (Supplementary Fig. 4c). Furthermore, the fraction of zymosan in LAPosomes per cell was stable upon Flag-ATG4BC78S expression, but not in Flag-ATG4Bwt transduced macrophages, which increased with time (Fig. 3H and Supplementary Fig. 4d). These results show that ATG4B plays a role during LAP and suggest that its oxidative inhibition by NOX2-produced ROS stabilizes LC3B conjugation to phagosomes during LAP.

### Oxidation of ATG4B during LAP

**Fig. 4:**
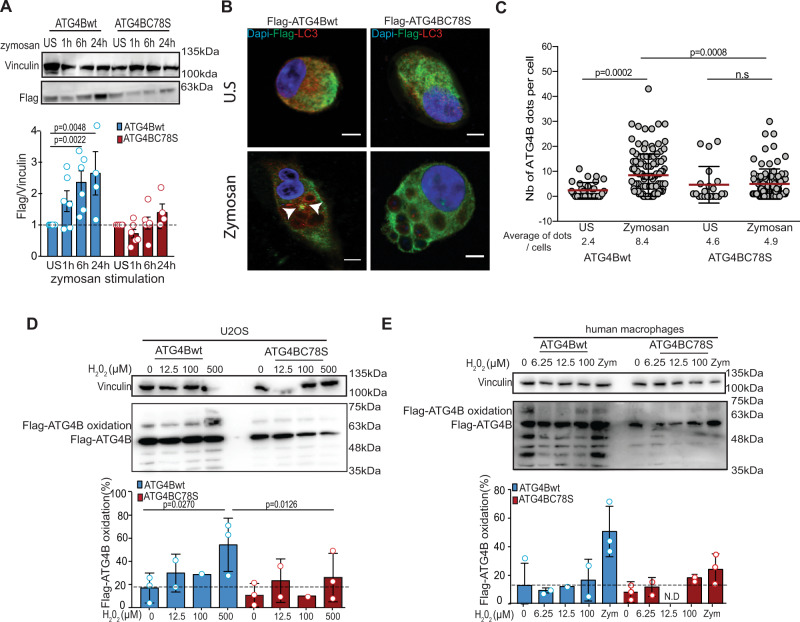
ATG4B is regulated by cysteine oxidation during LAP. **A** Macrophages transduced with lentiviruses encoding Flag-ATG4Bwt or Flag-ATG4BC78S were stimulated with zymosan for 1–24 h, lysed, and the level of Flag tagged proteins assessed by western blot. Bar graph shows the accumulation of Flag-ATG4Bwt upon LAP stimulation while no accumulation of the Flag-ATG4BC78S is observed. Bar graph represents mean ± SD of *n* = 6 independent experiments for the US to 6 h conditions and *n* = 4 independent experiments for the 24 h condition. Each symbol represents a single experiment. Mann–Whitney test, two-tailed. **B** Macrophages transduced with lentivirus expressing Flag-ATG4Bwt or Flag-ATG4BC78S were stimulated or not with zymosan for 6 h, fixed, and then stained for Flag (green) or LC3 (red). Representative images from three independent experiments are shown with scale bars of 5 µM. White arrows show Flag-ATG4B dots and US represents unstimulated conditions. **C** Graph shows the number of Flag-ATG4B dots per cells in unstimulated or zymosan stimulated conditions. Each dot represents a single cell and more than 20 cells per experiment were analyzed from three independent experiments. Unpaired Student’s *t* test, two-tailed. **D** U20S cells transduced with lentiviruses encoding Flag-ATG4Bwt or Flag-ATG4BC78S were treated with H_2_O_2_ and assessed for oxidation of ATG4B by PEG-switch assays. Bar graph shows quantification of Flag-ATG4B oxidation from treated U20S and bars represent mean ± SD of three independent experiments, and each symbol represents a single experiment. One-way ANOVA test. **E** Human macrophages transduced with lentiviruses encoding Flag-ATG4Bwt or Flag-ATG4BC78S were treated with H_2_O_2_ and assessed for oxidation of ATG4B by PEG-switch assays. Bar graph shows quantification of Flag-ATG4B oxidation from treated human macrophages and bars represent mean ± SD of three independent experiments, and each symbol represents a single experiment. N.D stands for not detected. Source data are provided as a Source Data file.

Protein thiols can serve as functional switches by undergoing rapid and reversible oxidative posttranslational modifications in response to redox changes in the environment^33^. ATG4 is a cysteine protease that can be inhibited by thiol oxidation. Interestingly, its oxidation sensitivity does not seem to pertain to its active site cysteine (Cys-74 in ATG4B), but instead to another cysteine residue, located in the vicinity of the active site (Cys-78), suggesting that oxidative modification of Cys-78, the nature of which is unknown (e.g., sulfenic acid formation, glutathionylation, intramolecular disulfide bond formation), leads to a structural change that prevents the enzyme from operating^30^. Surprisingly, in human macrophages overexpressing Flag-ATG4Bwt, an increase of Flag-ATG4Bwt protein levels were observed after LAP stimulation, while this protein accumulation was not observed in macrophages with the oxidation-insensitive mutant Flag-ATG4BC78S (Fig. 4A and Supplementary Fig. 5a). This result indicated that ATG4B accumulates during LAP and the associated ROS production. A study in the green alga *Chlamydomonas reinhardtii* showed that ATG4 oxidation leads to its aggregation^34^. Based on this study and our previous observations, we investigated if ATG4B aggregates during LAP by using immune fluorescence microscopy. Upon zymosan stimulation, Flag-ATG4Bwt formed more and bigger protein dots in macrophages compared to Flag-ATG4BC78S (Fig. 4B, C and Supplementary Fig. 5b). Indeed, oxidation-sensitive, but not oxidation-insensitive ATG4B formed protein aggregates in significantly more cells upon LAP stimulation (Fig. 4C). These results suggest that ROS production during LAP triggers ATG4B aggregation, inhibiting its LC3-delipidation activity. A role for Cys-78 in the oxidation of ATG4B in U2OS cells and macrophages was further suggested by the PEG-switch assay, a semiquantitative method to detect reversible cysteine oxidation^31,35^. In U2OS cells overexpressing Flag-ATG4Bwt and treated with H_2_O_2_, a shift in the molecular weight of Flag-ATG4Bwt was observed. Exposure to 500 µM H_2_O_2_ primarily induced one band with a molecular weight corresponding to 1X-PEGylated ATG4B, suggesting reversible oxidation of one cysteine (Fig. 4D). Formation of this band was significantly decreased upon mutation of Cys-78 (Fig. 4D). We speculate that the pattern of lower molecular weight bands was caused by degradation of Flag-ATG4B. ATG4B oxidation was also confirmed in macrophages upon H_2_O_2_ exposure and zymosan stimulation (Fig. 4E). Overall, these results supported the notion that LAP induces ATG4B oxidation and aggregation mainly in a Cys-78 dependent manner.

### Inhibition of ATG4B by ROS prolongs antigen presentation via MHC class II

**Fig. 5:**
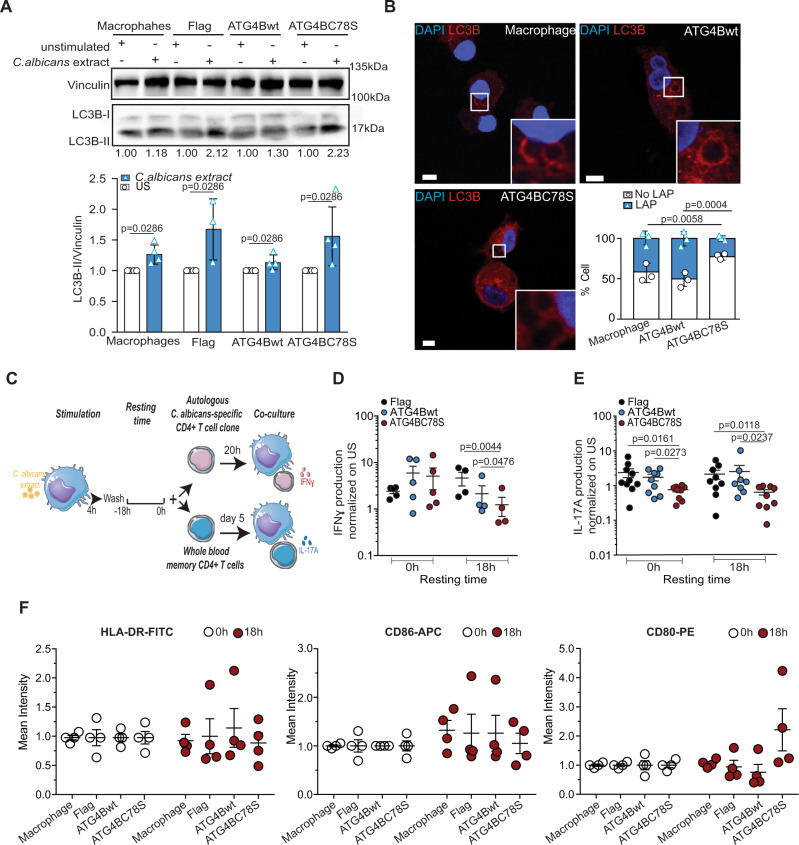
Inhibition of ATG4B-delipidation activity sustains MHC class II antigen presentation. Macrophages transduced with the indicated lentiviruses encoding Flag, Flag-ATG4Bwt, or Flag-ATG4BC78S were investigated. **A** Transduced macrophages were stimulated with *Candida (C.) albicans* extract for 6 h and the expression of LC3B was analyzed by western blot. Bar graph shows the level of LC3B-II expression normalized to vinculin. All conditions are normalized to each unstimulated condition (US), which is set to 1. Bars represent mean ± SD of three independent experiments, Mann–Whitney test, two-tailed. **B** Transduced macrophages were stimulated with *C. albicans* extract-coated beads for 6 h and then stained for LC3 (red). Representative images from three independent experiments, scale bars 5 µm, and inserts are zoomed 5× from white-framed regions of the original images. Bar graph shows the percentage of cells with or without LAPosomes. Bars represent the mean ± SD from three independent experiments, and each symbol represents a single experiment. One-way ANOVA test. **C** Experimental outline of MHC class II antigen-presentation assay. We created this diagram using the free online platform Smart Servier Medical Art. **D** ELISA assays on supernatants of a *C.* albicans-specific CD4^+^ T-cell clone cocultured with MHC class II matched macrophages transduced with the indicated Flag tagged constructs. Transduced macrophages were pulsed with *C. albicans* extract. Time indicated below the graph corresponds to the resting time of the pulsed macrophages before coculture. Scatter plot represents the IFNγ production normalized to unstimulated conditions (US) from five independent experiments and only values above the detection limit are displayed. Each symbol represents a single experiment. Ratio paired Student’s *t* test, two-tailed. **E** ELISA on supernatants of whole blood memory CD4^+^ T cells with autologous macrophages transduced with the indicated Flag tagged constructs. Transduced macrophages were pulsed with *C. albicans* extract. Time indicated below the graph corresponds to the resting time of the pulsed macrophages before coculture. Scatter plot represents the IL-17A production normalized to unstimulated conditions (US) from five independent experiments with five different donors and each symbol represents a biological replicate. Kruskal–Wallis test. **F** Macrophages transduced with the indicated lentiviruses were stimulated with *C. albicans* extract for 4 h. The macrophages were washed and then incubated for 18 h without stimuli or directly analyzed by FACS. Scatter plot from four independent experiments and each symbol represents a different experiment. Source data are provided as a Source Data file.

We previously reported that LAP is involved in prolonged MHC class II antigen presentation of phagocytosed *C. albicans* antigens^20^. Accordingly, we wondered whether the oxidative inhibition of ATG4B would support this prolonged MHC class II antigen presentation. For this purpose, human macrophages were transduced with lentiviruses encoding Flag, Flag-ATG4Bwt, or Flag-ATG4BC78S and were stimulated with *C. albicans* extract, also known to be a LAP stimulus. This stimulation indeed accumulated LC3B-II in human macrophages (Fig. 5A). However, Flag-ATG4BC78S expressing macrophages formed LAPosomes less frequently upon stimulation with *C. albicans* extract-coated beads, compared to their Flag-ATG4Bwt expressing counterparts (Fig. 5B). These data demonstrated that both *C. albicans* extract-coated beads and zymosan (Fig. 3G, H) require oxidation sensitivity of ATG4B to induce LAP. Therefore, we investigated the dependence of prolonged MHC class II antigen presentation after LAP, and especially the importance of oxidation sensitivity of ATG4B for this process. Along these lines, MHC class II antigen-presentation assays were performed according to the scheme in Fig. 5C. The IFNγ production of clonal *C. albicans-*specific CD4^+^ T cells of one donor in response to antigen-pulsed HLA-DR4 matched macrophages that expressed Flag, Flag-ATG4Bwt, or Flag-ATG4BC78S was assessed (Fig. 5D and Supplementary Fig. 6a). Oxidation-insensitive ATG4B expressing macrophages were significantly less able to present extracellular *C. albicans* antigens on MHC class II molecules to the specific CD4^+^ T-cell clone than their Flag or Flag-ATG4Bwt transduced counterparts (Fig. 5D). We also observed a nonsignificant decrease of antigen presentation upon Flag-ATG4Bwt expression, possibly due to saturation of the ROS regulation or a delipidation independent function of overexpressed ATG4B. In addition, the ability of whole blood memory CD4^+^ T cells from five independent donors, cocultured with their autologous macrophages pulsed with *C. albicans* extract, to secrete interleukin 17A (IL-17A) was also assessed (Fig. 5E and Supplementary Fig. 6b–e). The cytokine IL-17A is preferentially secreted by CD4^+^ Th17 cells in response to fungal infections, such as *C. albicans*^36^. Flag-ATG4BC78S expression was comparable to the expression levels of Flag-ATG4Bwt (Supplementary Fig. 6b, c). Transduced and antigen-pulsed macrophages expressing Flag or Flag-ATG4Bwt constructs were able to activate autologous *C.* albicans-specific whole blood memory CD4^+^ T cells to a similar extent. This was demonstrated by the secretion of IL-17A, as well as the production of the inflammatory Th17 cytokine IL-31 (Fig. 5E, Supplementary Fig. 6d, e). The differences in IL-17A production were established by ELISA and multiarray technology, while the latter was also used to detect the variation in IL-31. In the oxidation-insensitive ATG4B condition, with the compromised LAP pathway, the transduced and pulsed macrophages were less able to activate their autologous memory *C.* albicans-specific CD4^+^ T cells. Indeed, less IL-17A and IL-31 were secreted by the memory CD4^+^ T cells in coculture with the ATG4C78S transduced macrophages (Fig. 5E and Supplementary Fig. 6d, e). However, the overexpression of Flag, Flag-ATG4Bwt, and Flag-ATG4BC78S by macrophages only specifically influenced their ability to activate Th17 cells, as there were no significant differences in the secretion of promiscuously expressed human macrophage inflammatory protein-3α (MIP-3α or CCL20) (Supplementary Fig. 6e). Moreover, this inability of the ATG4C78S-macrophages to activate T cells was particularly pronounced at later time points (18 h) after antigen pulsing (Fig. 5D, E and Supplementary Fig. 6d, e). Altogether, these results suggest that the inhibition of ATG4B’s delipidation activity by oxidation during LAP leads to maintained MHC class II antigen presentation by human myeloid cells. Furthermore, we were wondering if this defect of presenting extracellular antigen on MHC class II molecules was associated with decreased MHC class II expression on the macrophage surface. However, we did not observe any difference in HLA-DR surface expression of macrophages after lentiviral transduction of Flag, Flag-ATG4Bwt, or Flag-ATG4BC78S (Fig. 5F and Supplementary Fig. 7a–d). Similarly, the expression of the two costimulatory molecules CD86 and CD80 was unaltered (Fig. 5F and Supplementary Fig. 7a–d), suggesting that the impairment of ATG4BC78S expressing macrophages to sustain MHC class II antigen presentation might be rather due to a defect in antigen loading during compromised LAP. Thereby, ROS production by NOX2 seems to inhibit LC3B deconjugation from LAPosomes via ATG4B oxidation and the resulting LAPosomes maintain endocytosed antigens for prolonged presentation on MHC class II molecules.

## Discussion

LAP shares some of its molecular machinery with autophagy but also has some unique requirements like ROS production^15,20^. In addition to the proposed role of NOX2-mediated ROS production during LAPosome formation, we highlight a role of NOX2 for its stabilization. Here, we report that NOX2-dependent ROS production at the pathogen-engulfing LAPosome inhibits ATG4B-mediated LC3B delipidation from the LAPosome membrane impeding its maturation. This seems to allow LAPosomes to efficiently deliver antigens for MHC class II restricted presentation to CD4^+^ T cells. Interestingly, we observed that the elevated and sustained oxidation level in close proximity to LAPosomes is linked to ATG4B oxidation and its aggregation, consistent with previous observations in the green alga *C. reinhardtii*^34^. Most likely, ATG4B will only be oxidized when in close proximity to the cytosolic LAPosome surface, since NOX-derived ROS entering the cytosol (i.e., H_2_O_2_ via aquaporins) can only travel short distances before being scavenged by the abundant thiol peroxidases^21^. Furthermore, it has been reported that cysteine 78 of ATG4B, located near its catalytic site, is the most redox-sensitive cysteine during autophagy^30^. Along these lines, we confirm that cysteine 78 also seems to be the most sensitive site for oxidation and inhibition of ATG4B, linked to LAPosome stabilization. By using ATG4BC78S, a mutant which is insensitive to oxidation, we demonstrated that uninhibited ATG4B efficiently removes LC3B from LAPosomes, thereby compromising MHC class II presentation of phagocytized antigen and impedes sustain LAP dependent antigen presentation by human macrophages. Our results link the NOX2 dependency of LAP to redox regulation of ATG4B, as the mechanism to stabilize LC3B on LAPosome membranes.

The regulation of phagocytosis by autophagy proteins plays a role at several levels for the immune response. LAP is crucial for antimicrobial immunity by promoting the fusion of pathogen-containing phagosomes with the lysosome. Indeed, LAP restricts *L. monocytogenes* infection in macrophages before they can escape from phagosomes. The β2 integrin Mac-1 was reported to mediate this cell intrinsic immune restriction of *L. monocytogenes*^37^. LAP has also been shown to be involved in the regulation of autoimmune diseases. The inefficient clearance of dying cells and apoptotic bodies by LAP was shown to enhance hyperinflammation, resulting in systemic lupus erythematosus^38^. Moreover, deficient MHC class II presentation of microbiota-derived outer membrane vesicles without LAP was also suggested to cause loss of regulatory CD4^+^ T cells and contribute to hyperinflammation in the gut^39^. Similarly, hyperinflammation in the tumor microenvironment in the absence of LAP promotes T-cell mediated immune control of cancers^40^. Thus, LAP promotes pathogen clearance and curbs hyperinflammation during innate immune responses. ATG proteins also regulate endocytosis, which in turn influences both MHC I and MHC II restricted antigen presentation to CD8^+^ and CD4^+^ T cells, respectively, and have an effect on the adaptive immune responses^3,41^. Consistent with these previous findings, our data suggest that NOX2-mediated inhibitory activity on ATG4B-delipidation contributes to sustaining LAP for more efficient MHC class II presentation. In addition, NOX2 play a role in delaying the acidification of phagosomes for enhanced antigen cross-presentation on MHC class I molecules to CD8^+^ T cells^42^.

Our findings also shed light on the membrane specificity of LC3 lipidation. Already earlier studies by Yoshinori Ohsumi’s laboratory using ATG4 deficient yeast, overexpressing the yeast LC3 orthologue ATG8, in its ATG4 processed form ATG8-I, indicated that ATG8 can be found on endosomes and vacuoles in addition to autophagosomes and that ATG4 is required to recycle ATG8^43^. In order to promote autophagosome biogenesis, ATG4 constitutively deconjugates ATG8 from all endomembranes to maintain a pool of unlipidated ATG8^44^. This suggested that LC3 lipidation is not selective to autophagic membranes, and probably can occur to some extend on all PI(3)P modified membranes. In this respect, we observed that in human macrophages the PI(3) kinase VPS34 was present at the pathogen-containing phagosome at the same time as LC3B, consistent with previous observations^15^. Furthermore, it was also shown that ATG1 (the yeast ULK1 orthologue) mediated phosphorylation of ATG4 inhibits ATG8 deconjugation at the forming autophagosome and is required to sustain ATG8 lipidation at this membrane, which is essential for autophagosome formation^23^. A recent study has also identified a new layer of regulation for LC3 deconjugation from liposomes. Indeed the phosphorylation of LC3C and GABARAPL2 by TBK1 impedes the binding and cleavage by ATG4 to maintain a unidirectional flow of autophagosomes to lysosomes^45^. In addition, our data describe a supportive role of ROS during LAP by inhibiting ATG4B-delipidation activity. Thereby, LC3 conjugation seems to occur at many more membranes than autophagosomes and LAPosomes, but inhibition of LC3 deconjugation by ATG4B at these sites allows the respective vesicles to use their LC3 decorated membranes to regulate their fate.

LAP contributes to cell intrinsic, innate, and adaptive immunity, and requires the inhibition of ATG4B-delipidation activity by NOX2 to sustain LC3B conjugation at pathogen-containing phagosome membranes allowing prolonged MHC class II antigen presentation by primary human macrophages. These beneficial features of LAP for immune responses could be harnessed in the future for instance by stimulation of the PI(3) kinase complex containing Beclin-1 with autophagy-inducing peptides for improved NOX2 assembly at phagosomal membranes, thereby stabilizing LAPosomes and promoting sustained MHC class II antigen presentation^46,47^.

## Methods

### Cell culture

Peripheral blood mononuclear cells (PBMCs) were isolated from leukocyte concentrates (Blood Donation Center Zürich) or from whole blood of healthy lab donors with the specific HLA-DRB1*0401 MHC class II haplotype by density gradient centrifugation using Ficoll-Paque (GE Healthcare). These studies were approved by the Cantonal Ethics Committee of Zurich, Switzerland (protocols no. KEK-StV-Nr.19/08 and 2019-00837). Research was conducted in accordance with the Declaration of Helsinki. The CD14^+^ cells were isolated from PBMCs by positive selection using magnetic beads (Miltenyi Biotec). Macrophages were grown in D10 (DMEM–GlutaMAX supplemented with 10% heat-inactivated fetal bovine serum (FBS; Sigma) and 1% penicillin/streptomycin (P/S; Gibco)). Recombinant human GM-CSF (1000 U/ml) was freshly added to the media at days 0, 3, and 5. Differentiated macrophages were used between days 6 and 9 of culture. The specific *C. albicans* CD4^+^ T-cell clone B3 used in this study was cultured as previously described^20^. Briefly, the CD4^+^ T cells were cultured with irradiated PBMCs and EBV transformed lymphoblastoid cell lines (LCLs) plus 1 µg/ml PHA-L in T-cell medium (RPMI supplemented with 10% heat-inactivated human serum, gentamycin, and 150 U/ml recombinant human IL-2). T cells were fed with irradiated LCLs and PBMCs in T-cell medium every week. Whole blood memory CD4^+^ T cells were isolated from the CD14-negative fraction by positive selection using magnetic beads (Miltenyi Biotec) and directly cocultured with their autologous macrophages in DMEM supplemented with 10% FBS and 1% P/S. The human osteosarcoma cell line U20S cells were cultured in D10 at 37 °C and 5% CO_2_.

### Lentiviral transduction and shRNA silencing

Lentiviral constructs carrying GFP-tagged LC3wt (pCSWG-LC3) and mRFP-tagged LC3Bwt were obtained as described respectively in Schmid et al. and Romao et al.^7,20^. Lentiviral constructs carrying GST-LC3B were a gift from Dr. Mathias Faure (Lyon, France)^48^. Flag-Flash-ATG4Bwt, Flag-Flash-ATG4BC78S, and the Flag control were synthetized by GeneArt, Thermo Scientific. The different ATG4B genes were first subcloned in pENTR1A noccDB gene vector (Addgene) and finally introduced into the pLENTI-CMV-Puro DEST vector (Addgene) by site-specific recombination (Gateway Cloning technology, Thermo Fisher Scientific). Lentiviral particles were produced as described in Schmid et al.^7^. Briefly, lentiviral particles were produced by cotransfecting the lentiviral vector DNA together with two helper plasmids, pCMV∆R8.91 and pMDG, into HEK293T cells by calcium phosphate transfection. At day 2 post transfection, the supernatant containing the recombinant lentiviral particles was collected, filtered through a 0.45 µM filter, and concentrated overnight in Peg-IT solution (System Biosciences). Concentrated lentiviral particles were kept at −80 °C. The primary human macrophages were used between days 5 and 7, transduced with the respective lentiviruses. For transduction, the lentivirus particles were added together with 6 µg/ml polybrene (EMD Millipore) to the macrophages and centrifuged at 1900 × *g* for 45 min at 37 °C. Twenty-four hours after transduction, the culture medium was replaced, and the cells were incubated for another 24 h.

### LAP stimuli and antibodies

Differentiated macrophages were stimulated with different LAP stimuli: 50 µg/ml of uncoupled zymosan (InvivoGen), 50 µg/ml Texas Red zymosan (Invitrogen), and 40 µg *C. albicans* protein extract or beads coated with *C. albicans* protein extract at a ratio 10:1 (bead:macrophage). To synchronize the LAP stimulation, the cells were centrifuged 500 × *g* during 5 min and then incubated at 37 °C, 5% CO_2_. Antibodies and cell dyes that were used in this study are listed in Supplementary Table 1.

### Beads coated with LAP stimuli

The beads were coated as described in Romao et al.^20^. Briefly, 20 µg of *C. albicans* protein extract was added to 1 × 10^8^ polystyrene fluorescence blue beads (Sigma, L0280) in PBS buffer and incubated for 1 h at RT and then overnight at 4 °C with continuous rotation. Coated beads were washed with PBS, counted and immediately used.

### Labeling of zymosan with OxyBURST

Zymosan was labeled with amine-reactive OxyBURST-Green H2DCFDA (Molecular Probes) using an adapted protocol from Dingjan et al.^49^. Briefly, 10 mg of zymosan was boiled and then washed with 0.1 M Na_2_CO3, pH 8.3, between washes sonication rounds were performed. Then, zymosan was incubated 1 h at RT with 2.5 mg/ml OxyBURST-Green. Subsequently, the OxyBURST-Green was activated by adding 1.5 M hydroxylamine, pH 8.5. Excess of hydroxylamine and unbound probes were removed by a series of washes with a decrease of the concentration of DMSO-PBS. Finally, zymosan-OxyBURST was resuspended at a final concentration of 50 mg/ml and directly used to stimulate human macrophages at a final concentration of 100 µg/ml and then used for confocal microscopy analysis.

### Immunofluorescence microscopy

Overall, 5 × 10^5^ CD14^+^ cells were plated on sterile glass cover slides coated with Poly-L-Lysine (Sigma) and differentiated into macrophages. The cells were fixed with 4% paraformaldehyde for 15 min at RT and then permeabilized with PBS-Triton 100 × 0.1% for 5 min. Unspecific sites were blocked with PBS-FBS 1% for 1 h at RT, and then the cells were stained for 1 h at room temperature. Primary and secondary antibodies were diluted in PBS-FBS 1%. Before mounting the cover slides, the cells were stained with DAPI for 5 min. Slides were visualized using a light confocal microscopy (LEICA, SP8 upright).

### Quantitative analysis of immunofluorescence images

All the images were independently analyzed with the Fiji software. At least 25 nuclei from at least three independent experiments were analyzed. One or two investigators performed the quantification manually and blindly.

Zymosan-OxyBURST intensity level was evaluated by first selecting a phagosome or LAPosome based, respectively, on morphology and LC3B recruitment. The areas corresponding to phagosomal membranes were then selected, and finally the intensity was measured. All images were evaluated independently. At least 25 phagosomes for at least three independent experiments each were analyzed by two investigators.

Quantification of ATG4B dots per cell was performed using a semiautomatic pluging designed on Fiji J (Supplementary Fig. 5b). For every image the channels were separated and then a particle analysis was performed in the ATG4B channel (using maximum intensity with a manual threshold) and used to create a mask identifying objects (ATG4B dots, size exclusion was set up to 5–30 pixel), followed by quantification.

### PEG-switch assay

Reversible cysteine oxidation assays were performed as previously described using the PEG-switch assay^2,31,35^. Briefly, transduced U20S or macrophages with Flag-ATG4Bwt or Flag-ATG4BC78S were stimulated with H_2_O_2_ (6.25–500 µM) for 1 h or with zymosan 6 h. Treated and untreated cells were lysed for 15 min into alkylating buffer (1% SDS, 100 mM Tris, pH 7.4, and 100 mM maleimide) and then incubated at 50 °C under vigorous agitation for 25 min. After incubation, samples were desalted using Zeba Spin Desalting Columns. Two hundred nanomolar of reducing agent dithiothreitol was added to the samples to quench the maleimide and reduce reversible oxidation. After 20 min of incubation at RT, the samples were desalted using Zeba Spin Desalting Columns. Labeling buffer (10 mM PEG-maleimide 5000, 1% SDS, and 100 mM Tris, pH 7.4) was added to each sample and incubated at RT for 2 h. Finally, samples were prepared for SDS-PAGE gel electrophoresis by addition of sample buffer containing 5% of beta-mercaptoethanol and immunoblotted as described.

### Western blot analysis

Human macrophages were lysed in Laemmli buffer, then centrifuged at 16 × *g* at 4 °C for 15 min. Protein samples were boiled for 5 min at 95 °C, separated by SDS-PAGE (10 or 12.5% bis-acrylamide) and transferred to PVDF membranes. The membranes were blocked with PBS-5% nonfat milk, incubated overnight with the primary antibody, then for 1 h with secondary antibody, and finally visualized, with ECL detection kit (WesternBright Sirius femtogram HRP Substrate Witec AG7002325). Quantitative analysis of blotting signals was performed using Image J Software. For presentation of full scan blots, see the Supplementary Figs. 8 and 9.

### Antigen-presentation assays using a *C. albicans*-specific CD4^+^ T-cell clone

Human macrophages were generated from specific HLA-DRB1*0401^+^ healthy donors, and the HLA-DRB1*0401 restricted autologous *C. albicans*-specific CD4^+^ T cells clone B3 was used as described in the study of Romao et al.^20^. The specific macrophages were transduced with lentiviruses carrying Flag, Flag-wild-type ATG4B, or Flag-ATG4B mutants for 24 h and then pulsed with 40 µg/ml of soluble *C. albicans* extract. After 4 h of stimulation, the cells were washed and new medium was added (RPMI 1640 + 5% of FBS and gentamycin). After 0 and 18 h, B3 CD4^+^ T cells were added for coculture at a ratio of 1.75:1 for 20 h.

### Antigen-presentation assays using whole blood memory CD4^+^ T cells

Human macrophages and autologous whole blood memory CD4^+^ T cells were purified from healthy donors. The macrophages were transduced with lentiviruses carrying Flag, Flag- ATG4Bwt, or Flag-ATG4BC78S mutant for 24 h and then pulsed with 80 µg/ml of soluble *C. albicans* extract. After 4 h of stimulation, the cells were washed and new medium was added (DMEM + 10% of FBS and 1% P/S). Immediately or after 18 h of resting time, the purified whole blood memory CD4^+^ T cells were added for coculture at a ratio of 2:1 for 5 days.

### MHC class II and costimulatory molecule surface expression

For FACS nonspecific binding was blocked using human TruStain FcX (BioLegend). Macrophages were stained with Zombie aqua, CD3-APC-Cy7 clone HIT3a CD86-APC and PE-Cy5 clone IT2.2, HLA-DR-FITC and APC clone L243 (BioLegend), CD14-BV650 and BUV737 clone M5E2, and CD80-PE clone L307.4 (BD Pharmingen^TM^) and then acquired by flow cytometry. The FACS data were analyzed with FlowJo software (TreeStar Inc.).

### Cytokine assays

For IFNγ cytokine detection, supernatants were harvested after 20 h of coculture of clonal B3 T cells with antigen-pulsed macrophages (1.75:1), diluted and directly plated into precoated 96-well ELISA plates (Nunc-Immuno MaxiSorp; Thermo Fisher Scientific). The cytokine detection from the supernatants of cocultures between memory CD4 ^+^ T cell and their autologous macrophages (2:1) were harvested after 5 days. Supernatants were directly distributed into precoated 96-well ELISA plates. IL-17A was detected using the Human IL-17A Quantikine High Sensitive ELISA Kit (R&D). Recombinant human IFNγ and IL-17A were used to determine the respective standard curves. Supernatants were in addition used for multiarray assays, using the V-PLEX Th17 Panel 1 Human Kit (MSD) according to the manufacturer’s recommendations.

### Quantification and statistical analysis

Number of experimental repeats is specified in the corresponding figure legends for each type of experiment. For statistical analysis, data were subjected to paired Student’s *t* tests for ELISA, one-way ANOVA, unpaired Student’s *t* test, or Mann–Whitney test for immunofluorescence and western blot. *p* values of less than 0.05 were considered significant.

### Reporting summary

Further information on research design is available in the Nature Research Reporting Summary linked to this article.

## Supplementary information

Supplementary Information

Reporting Summary

## Data Availability

All the data generated or analyzed during this study are included in this published article and its supplementary information files, or are available from the corresponding author upon reasonable request. Source Data are provided with this paper.

## Source data

Source Data
