# Supplementary material for: Oxidation inhibits autophagy protein deconjugation from phagosomes to sustain MHC class II restricted antigen presentation
Source: Nat Commun. 2021 Mar 8;12:1508. doi: 10.1038/s41467-021-21829-6 (PMC7940406; doi:10.1038/s41467-021-21829-6)
Supplement: Supplementary file 1 — Supplementary Information [file 41467_2021_21829_MOESM1_ESM.pdf]

Oxidation inhibits autophagy protein deconjugation from phagosomes to sustain MHC class II restricted antigen presentation

Laure-Anne Ligeon[1], Maria Pena-Francesch[1], Liliana Danusia Vanoaica[1,] Nicolás Gonzalo Núñez[2], Deepti Talwar[3,4], Tobias P. Dick[3,4] and Christian Münz[1]*

Supplementary Information

Supplementary Table 1: Antibodies and cells dyes

Reagent	Source	Identifier	Dilution /Concentration
**Antibody**			
Mouse anti-ATG4B	MBL	M134-3	IF 1:50<br>WB 1:500
Rabbit anti-ATG16L1	Cell signaling	2000 -10S	IF 1:100
Rabbit anti-ATG12	Cell signaling	D6D5	IF 1:100
Rabbit anti-ATG14	Proteintech	19491-1-AP	IF 1:100
Mouse anti-EEA1	BD	610457	IF 1:100
Mouse anti-GP91	Santa Cruz	SC-130543	IF 1:100
Mouse anti-p22-phox	Santa Cruz	SC-130550	IF 1:50
Mouse anti-p40-phox	Santa Cruz	SC-48388	IF 1:50<br>WB 1:500
Mouse anti-LAMP1	Santa Cruz	SC-18821	IF 1:100
Rabbit anti-LC3B	MBL	PM036	IF 1:100
Rabbit anti-LC3B	Novus Biologicals	NB100-2220	WB 1:1000
Rabbit anti-VPS34	Sigma	K3141	IF 1:100<br>WB 1:1000
Rat anti-Flag	Biolegend	637303	IF 1:100<br>WB 1:1000
Mouse anti GST	Cell signaling	26H1	IF 1:100<br>WB 1:1000
Mouse-Vinculin	Sigma	V9131	WB 1:10000
Mouse Actin-HRP	Abcam	Ab49900	WB 1:10000
Mouse anti HRP	Jackson ImmunoResearch		WB 1:10000
Rabbit anti HRP	Jackson		WB 1:10000

	ImmunoResearch		
Goat AlexaFluor 488	Invitrogen		IF 1:500
Goat AlexaFluor 555	Invitrogen		IF 1:500
Goat AlexaFluor 647	Invitrogen		IF 1:500
DAPI	Invitrogen	D3571	IF 1:5000
**Chemicals**			
VPS34-IN1 (SAR405)	selleckchem	S7682	100µM
Diphenyleneiodonium (DPI)	Sigma Aldrich	D2926	50nM
Apocynin	Sigma Aldrich	W508454	50nM
Bafilomycin	Sigma Aldrich	B1793	10µM
Hydrogen peroxide solution H_20_2	Merck	H1009	6.25-500µM
Rapamycin	Sigma Aldrich	553210	20µM
OxyBURST	Invitrogen	D2935	
Zymosan Texas Red	Invitrogen	Z2843	100µM
Zymosan	Invivogen	tlrl-zyn	100µM
Blue beads	Sigma	L0280	
Zeba™ Spin Desalting Columns, 7K MWCO, 2 ml	ThermoFisher	89890	
Maleimide	Sigma Aldrich	129585	
PEG-Maleimide (Metoxi-PEG-Maleimide)	Sigma Aldrich	63187-	
**Software**<br>ImageJ and Fiji<br>Prism			

Supplementary Figure 1

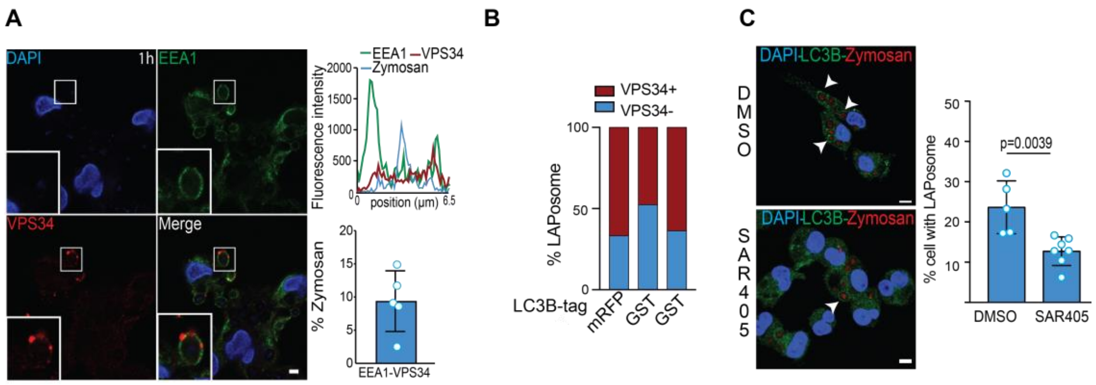

VPS34 and EEA1 localize to zymosan-containing phagosomes. (A) Human macrophages were stimulated with zymosan for 1 h, fixed and co-stained for EEA1 (green) and VPS34 (red). EEA1 and Vps34 fluorescence intensities were quantified along the white segment in the merged fluorescence panel and plotted as a histogram. Scale bar is 5 µm, and inserts are zoomed 5X from white-framed regions of the immune fluorescence micrographs. Bar graph shows quantification of percentage of zymosan decorated with both EEA1 and VPS34. Bar represents the mean ± SD of data pooled from 5 independent experiments and each symbol represents a single experiment. **(B)** Bar graph shows the percentage of LAPosomes decorated with VPS34 or not quantified for each experiment pooled in Figure 1B. Each bar represents a single experiment, n=3. **(C)** Macrophages were pre-treated with a VPS34 inhibitor (SAR405), stimulated with zymosan Texas Red for 6 h, fixed and stained for LC3B (green). White arrows point to LAPosomes and scale bars indicate 5 µm. Bar graph shows the percentage of cells with LAPosomes in DMSO or SAR405 treatment condition. Bar represents the mean ± SD of data pooled from at n= 5 (DMSO) and n=7 (SAR405) independent experiments, and each symbol represents a single experiment; unpaired student t test, two-tailed. Source data are provided as a Source Data file.

Supplementary Figure 2

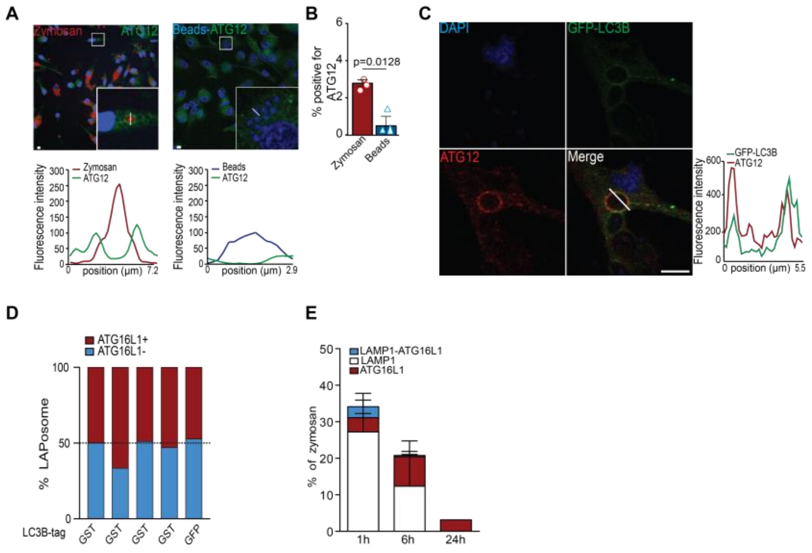

ATG12 and ATG16L1 persist for prolonged time periods on zymosan-containing LAPosomes. (A) Macrophages were stimulated with zymosan-TexasRed (red) or with inert beads (blue) for 6h and immunostained for ATG12 (green). Histograms showed the fluorescence intensity of ATG12 along the white segment in the merged fluorescence panel. Image from one representative experiment over three independent experiments, the scale bar is 5 µm, and inserts are zoomed 5X from white-framed regions of the immune fluorescence micrographs. **(B)** Quantification of the percentage of zymosan or beads positive for ATG12 is shown in the bar graph. Bars represent the mean of three independent experiments quantified by two independent investigators. Each symbol represents an individual experiment; unpaired student t test, two-tailed. **(C)** Human macrophages transduced with lentiviruses carrying the GFP-LC3B (green) reporter gene were stimulated with zymosan for 6 h and stained for ATG12 (red). Image from one representative experiment over three independent experiments, the scale bar is 5 µm, and inserts are zoomed 5X from white-framed regions of the immune fluorescence micrographs. Histogram shows the fluorescence intensity of GFP-LC3B and ATG12 along the white segment in the merged fluorescence image. **(D)** Bar graph shows the percentage of LAPosomes decorated with ATG16L1 quantified for each experiment pooled in Figure 1E. Each bar represents a single experiment, n=5. **(E)** Bar graph shows the means ± SEM percentage of zymosan-containing phagosomes single positive for ATG16L1 or LAMP1 or both after 1, 6 or 24h of incubation. Bars represent the mean of three independent experiments. Source data are provided as a Source Data file.

Supplementary Figure 3

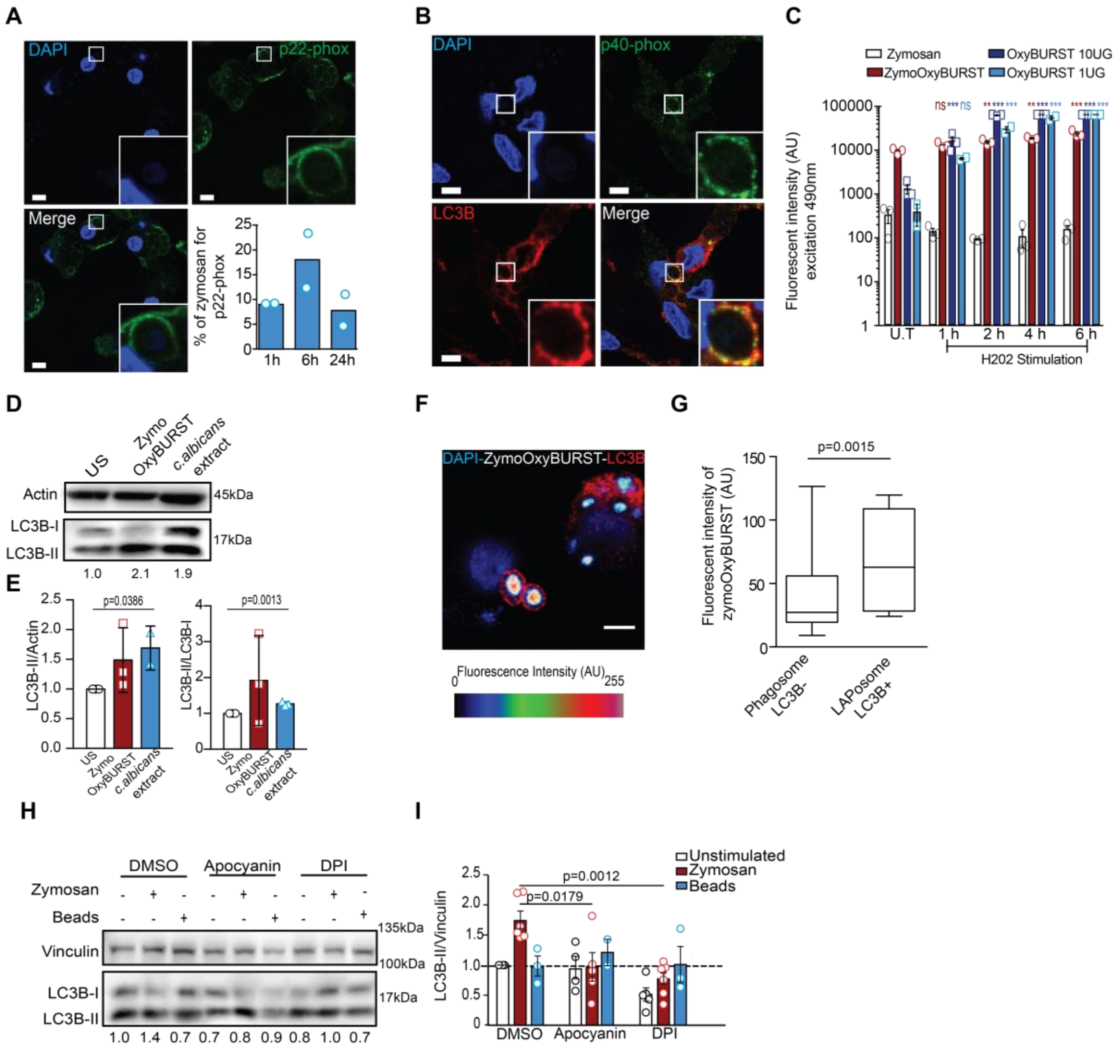

NOX2 components and ROS production can be found at LAPosomes. (A) Human macrophages were stimulated with zymosan for 6h, fixed and co-stained for p22-phox (green). Image from one representative experiment over three independent experiments. Scale bars indicate 5 µm, and inserts are zoomed 5X from white-framed regions of the original images. Bar graph shows the percentage of zymosan displaying p22-phox after the indicated incubation times. Bars are means of two independent experiments and each symbol represents a single experiment. **(B)** Human macrophages were stimulated with zymosan for 6 h, fixed and co-stained for p40-phox (green) and LC3B (red). Image from one representative experiment over three independent experiments. Scale bars indicate 5 µm, and inserts are zoomed 5X from white framed regions of the original images. **(C)** Fluorescence intensity of zymosan, OxyBURST-coated zymosan (zymoOxyBURST) or free OxyBURST 1µg and 10µg measured after 1h up to 6h of H_20_2 stimulation. Bars are means ± SD of three independent experiments. One-way ANOVA statistics: **, p < 0.005; *** p <

0.0005. (**D-E**) Human macrophages were stimulated with OxyBURST-coated zymosan (zymoOxyBURST) or *Candida albicans* extract for 6h, lysed and the change of LC3B protein levels assessed by Western blotting. Immunoblots were quantified by densitometry and quantitative data is shown in the bar graph. The left graph shows the means of LC3B-II bands intensity normalized to the loading control, and the right graph shows the means of the LC3B-II/LC3B-I ratio. Bars are means ± SD of three independent experiments and each symbol represents a single experiment. All conditions are normalized to the unstimulated condition (US), which is set to 1. T-test, two-tailed. **(F)** Macrophages were stimulated with zymo-OxyBURST for 6h, fixed and stained for LC3. Image from one representative experiment over three independent experiments. Image represents the maximal projection of z-stack. Scale bars indicate 5 µm. **(G)** OxyBURST intensity level was measured inside LAPosomes and LC3 negative phagosomes on maximal projection of z-stack. Box represents the min to max of two independent experiments and for each more than 20 cells were analyzed. unpaired student t test, two-tailed. **(H)** Macrophages pretreated with NOX inhibitors were stimulated with zymosan or beads for 6h and then lysed. Cell lysates were subjected to SDS-page gel electrophoresis and immunoblotted for LC3B and the loading control vinculin. One representative experiment of three is shown. **(I)** Bar graph shows the level of LC3B-II protein expression normalized to vinculin. All conditions are normalized to the DMSO unstimulated condition, which is set to 1. Bars represent the mean ± SD: DMSO (n=7 US, n=6 Zym, n=3 Beads), Apocyanin (n=4 US, n=5 Zym, n=2 Beads) and DPI (n=5 US, n=7 Zym, n=3 Beads) independent experiments, and each symbol represents a single experiment, Unpaired t-test, two-tailed. Source data are provided as a Source Data file.

Supplementary Figure 4

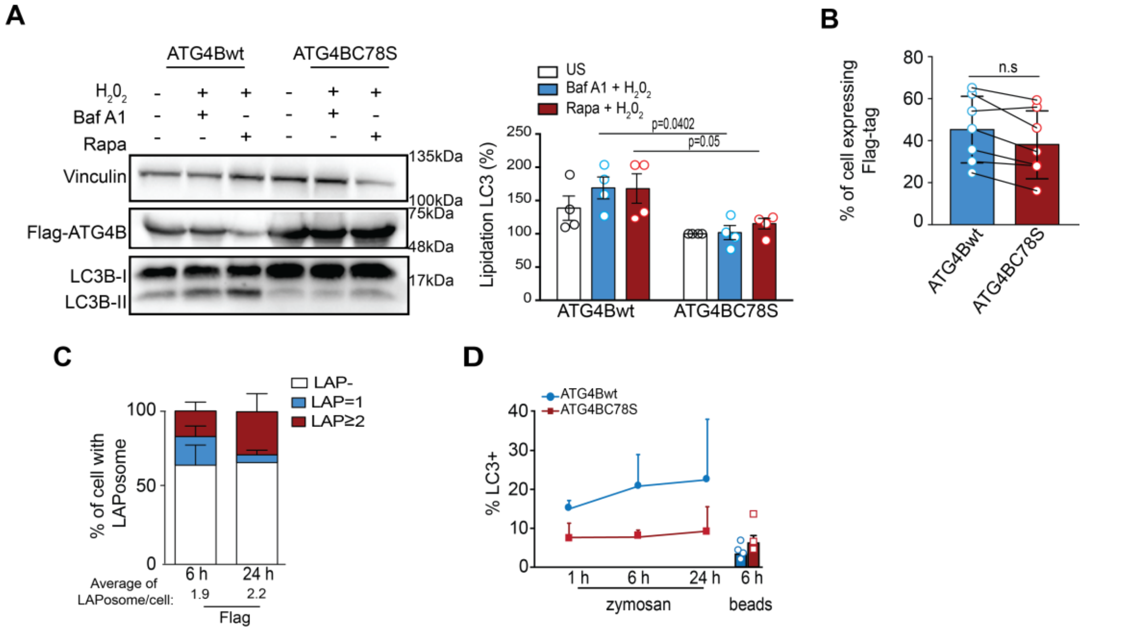

ROS increase lipidated LC3B levels and oxidation insensitive ATG4B compromises LAPosome formation. (A) Macrophages transduced with the indicated lentiviruses encoding Flag-ATG4Bwt or Flag-ATG4BC78S were treated with rapamycin (Rapa, 20µM), bafilomycinA1 (BafA1, 10µM) and with H_2O_2 (0.5 U). Lysed samples were assessed by Western Blot for LC3 and Flag protein levels. Bar graph shows the levels of LC3B-II expression normalized to the loading control vinculin. All conditions are normalized to Flag-ATG4BC78S unstimulated (US) conditions, which is set to 1. Bars are means ± SD of four independent experiments and each symbol represents a single experiment. One-way ANOVA test. (B) Bar graph shows the percentage of cells transduced with Flag-ATG4Bwt or Flag-ATG4BC78S. Each dot represents a single experiment (n=7) and conditions of individual experiments are connected with lines. Unpaired t-test, two-tailed; non-significant (n.s). **(C)** Bar graph shows the percentage of LAPosomes formed per cell upon zymosan stimulation for 6h or 24h in macrophages overexpressing the Flag control. Bars of the graph represent the mean ± SEM of 3 independent experiments. **(D)** Graph shows the percentage of LC3B positive zymosan-containing phagosomes and beads positive for LC3B in macrophages transduced with Flag-ATG4Bwt or Flag-ATG4C78S at the indicated incubation times. Graph shows the means ± SEM of 3 independent experiments. Source data are provided as a Source Data file.

Supplementary Figure 5

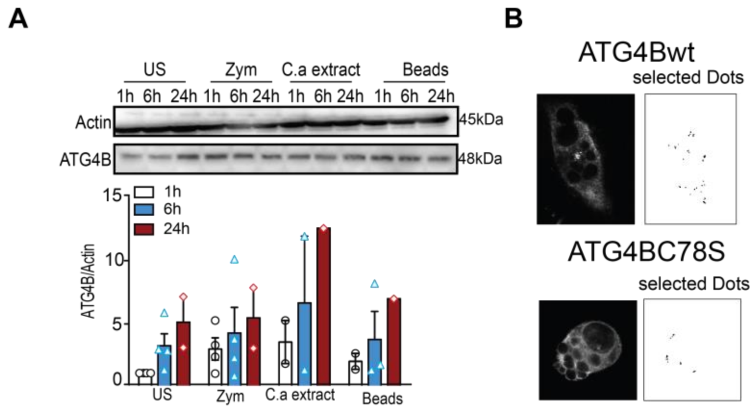

ATG4 accumulates upon LAP stimulation. (A) Macrophages were stimulated with zymosan, *Candida albicans* extract or inert beads for 1h to 24h, lysed and the level of ATG4B proteins assessed by Western blot. Bar graph shows the accumulation of ATG4B upon LAP stimulation. Bar graph represents means ± SD of independent experiments; each symbol represents a single experiment. n= 4 for unstimulated and zymosan at 1h and 6h, and n=2 for 24h time point, n=2 for C.a and beads condition for time point 1h and 6, n=1 for 24h **(B)** Representative image used in Figure 4B and masks obtained as described in the methods to count the number of Flag-ATG4B dots per cell. Source data are provided as a Source Data file.

Supplementary Figure 6

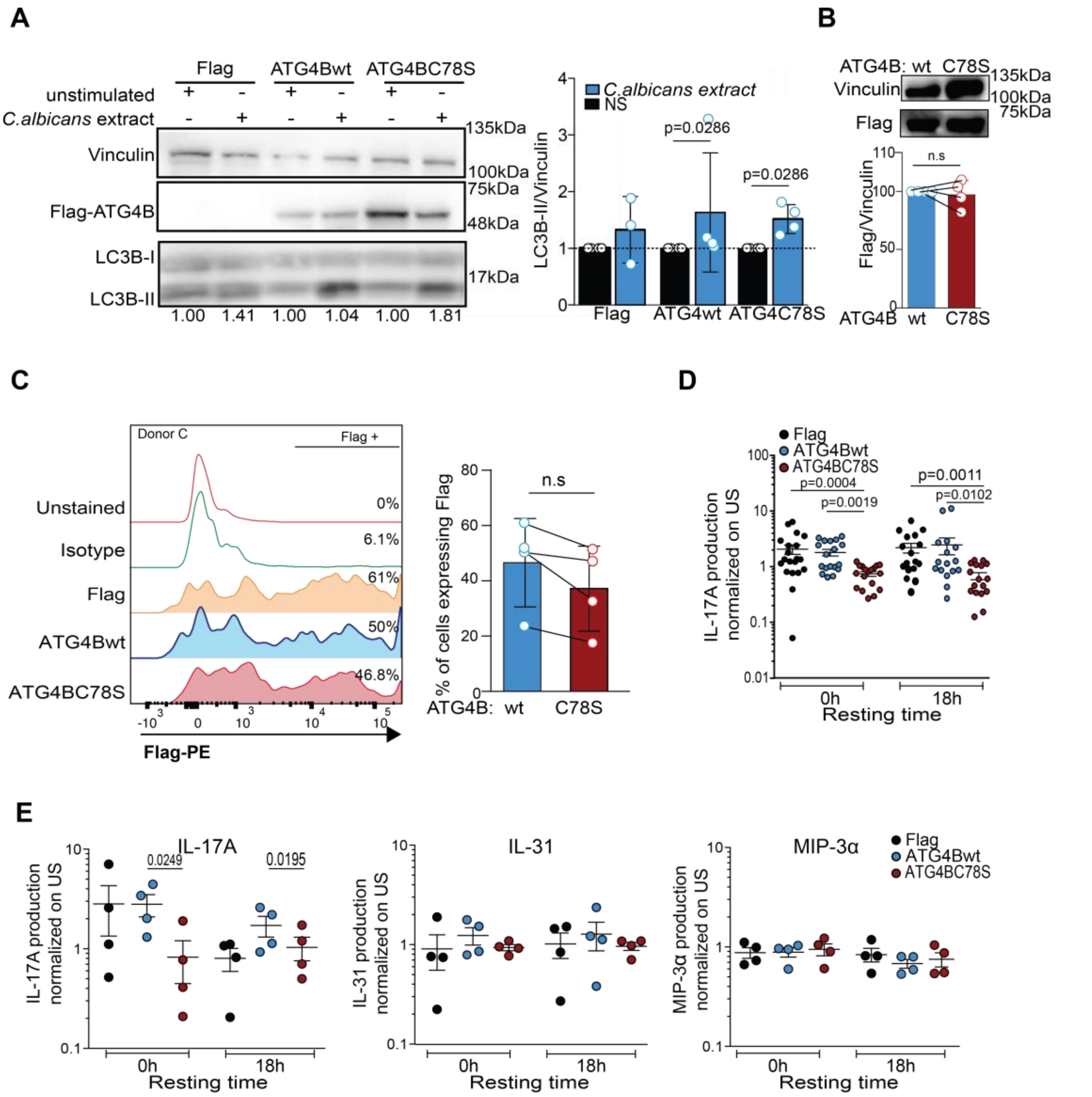

Oxidation-insensitive ATG4B blocked LAPosome stabilization and impedes sustained antigen presentation via MHC class II molecules. Macrophages transduced with the indicated lentiviruses were stimulated with *Candida albicans* extract for 4h. **(A)** Transduction and stimulation by *Candida albicans* extract of the macrophages used for the IFNγ EILSA were checked by Western blot and showed accumulation of LC3B-II. Bar graph shows means ± SD of three (Flag) or four (ATG4B constructs) independent experiments for the indicated conditions. Each symbol represents a single experiment Mann-Whitney test, two-tailed. (B-C) Percentage of transduction of Flag-ATG4Bwt or Flag-ATG4BC78S from the macrophages used in the Figure 5E and S6D-E. (**B**) Western blot shows a representative experiment out of 4 independent experiment (n=4 different donors). Bar graph shows the means ± SD of the level of Flag expression normalized to the

loading control vinculin. All conditions are normalized to their Flag-ATG4Bwt unstimulated (US) conditions, which is set to 100. Each symbol represents a single experiment from a single donor and conditions of individual experiments are connected with lines, Mann-Whitney test, two-tailed: n.s, non significant. (**C**) Flow cytometry analysis of Flag expression (filled histogram) within the macrophages used in Figure 5E and S6D-E and the corresponding control (open histogram). Representative histogram from four independent experiments. Bar graph shows the means ± SD of the percentage of cells expressing Flag and each symbol representing an independent experiment from a single donor. Conditions of individual experiments are connected with lines. Unpaired t-test, two-tailed: n.s, non significant (**D**) ELISA on supernatants of whole blood memory $CD4^+$ T cell co-cultured with autologous macrophages transduced with the indicated Flag tagged constructs. Transduced macrophages were pulsed with *Candida albicans* extract. Scatter plot shows the means ± SD represents five independent experiments from five different donors used in the Figure 5E. Each symbol represents a technical replicate. One-way ANOVA test (**E**) Cytokine production (IL-17A, IL-31 and MIP-3α) from the supernatants of pulsed and transduced macrophages co-cultured with autologous whole memory $CD4^+$ T cells for 5 days by multi-array analysis. Scatter plot represents the cytokine production from 4 independent experiments with 4 different donors and each symbol represents an individual donor, Kruskal-Wallis test. Source data are provided as a Source Data file.

Supplementary Figure 7

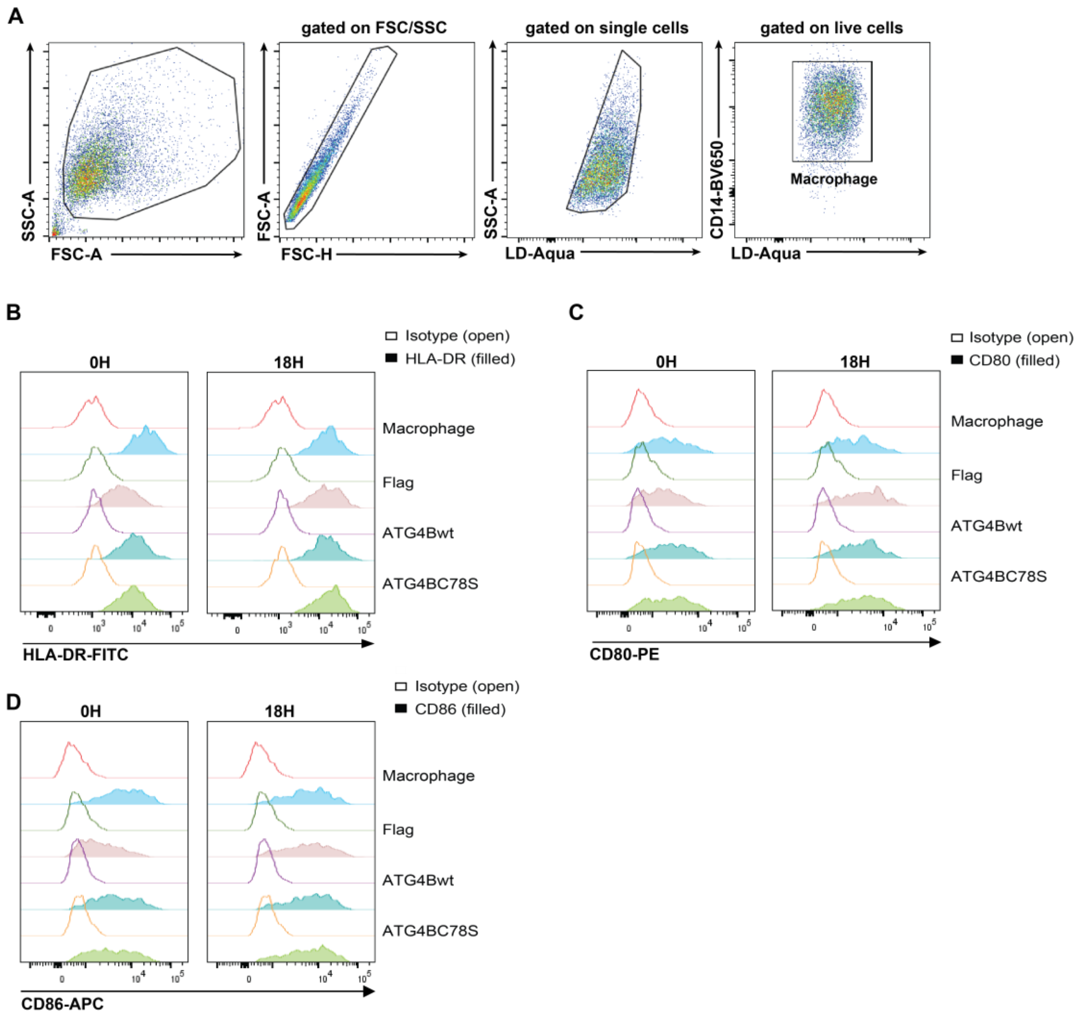

ROS insensitive ATG4 does not affect MHC class II and co-stimulatory molecule expression on human macrophages. Macrophages transduced with the indicated lentiviruses were stimulated with Candida albicans extract for 4h. The macrophages were washed and then incubated for 18h without stimuli or directly analyzed by FACS. (A) Gating strategy to determine HLA-DR, CD80 and CD86 expression on transduced macrophages. (B-D) Flow cytometric analysis of HLA-DR (B), CD80 (C) and CD86 (D) expression (filled histogram) by macrophages and the corresponding isotype control (open histogram). Representative histograms from three independent experiments.

Supplementary Figure 8

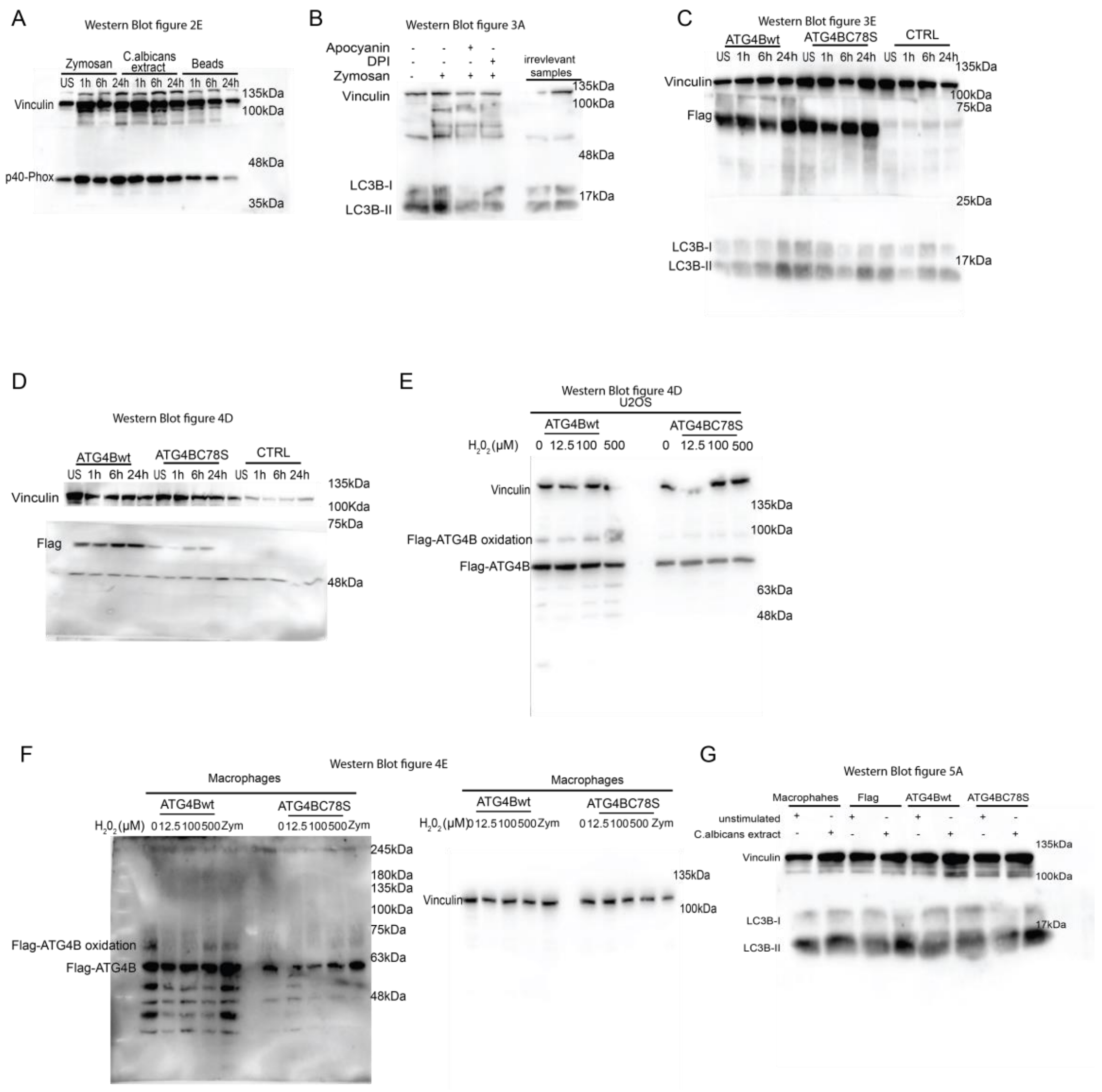

Full scans of the western blots presented in Figures 2 to 5.

Supplementary Figure 9

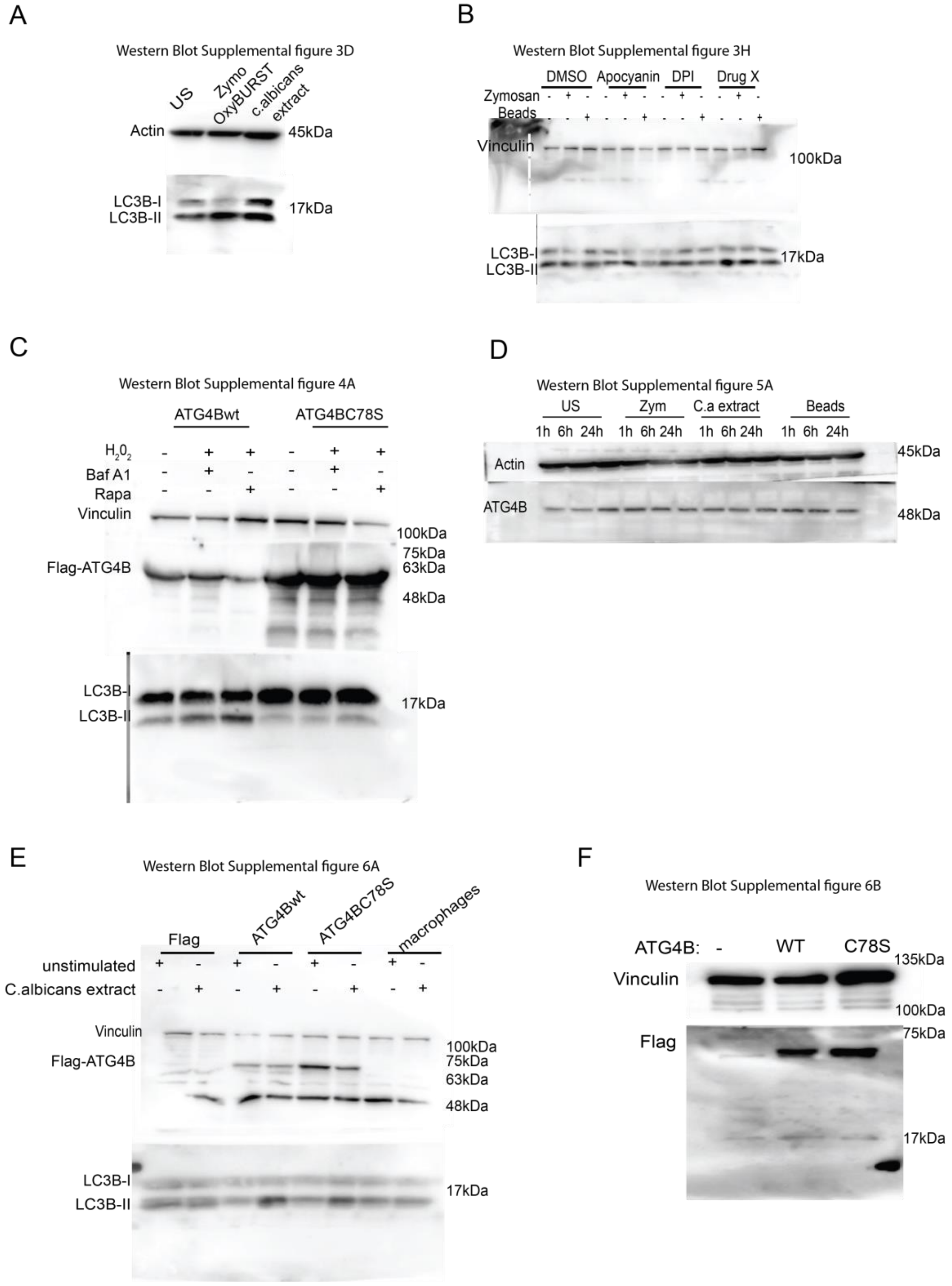

Full scans of the western blots presented in Supplementary Figures 2 to 5.
