# Supplementary material for: Oxidation inhibits autophagy protein deconjugation from phagosomes to sustain MHC class II restricted antigen presentation
Source: Nat Commun. 2021 Mar 8;12:1508. doi: 10.1038/s41467-021-21829-6 (PMC7940406; doi:10.1038/s41467-021-21829-6)
Supplement: Supplementary file 2 — Reporting Summary [file 41467_2021_21829_MOESM2_ESM.pdf]

nature research

Corresponding author(s): Christian Münz

Last updated by author(s): Feb 9, 2021

Reporting Summary

Nature Research wishes to improve the reproducibility of the work that we publish. This form provides structure for consistency and transparency in reporting. For further information on Nature Research policies, see our Editorial Policies and the Editorial Policy Checklist.

Statistics

For all statistical analyses, confirm that the following items are present in the figure legend, table legend, main text, or Methods section.

n/a	Confirmed	
☐	☒	The exact sample size (n) for each experimental group/condition, given as a discrete number and unit of measurement
☐	☒	A statement on whether measurements were taken from distinct samples or whether the same sample was measured repeatedly
☐	☒	The statistical test(s) used AND whether they are one- or two-sided<br>*Only common tests should be described solely by name; describe more complex techniques in the Methods section.*
☒	☐	A description of all covariates tested
☐	☒	A description of any assumptions or corrections, such as tests of normality and adjustment for multiple comparisons
☐	☒	A full description of the statistical parameters including central tendency (e.g. means) or other basic estimates (e.g. regression coefficient) AND variation (e.g. standard deviation) or associated estimates of uncertainty (e.g. confidence intervals)
☐	☒	For null hypothesis testing, the test statistic (e.g. F, t, r) with confidence intervals, effect sizes, degrees of freedom and P value noted<br>*Give P values as exact values whenever suitable.*
☒	☐	For Bayesian analysis, information on the choice of priors and Markov chain Monte Carlo settings
☒	☐	For hierarchical and complex designs, identification of the appropriate level for tests and full reporting of outcomes
☒	☐	Estimates of effect sizes (e.g. Cohen's d, Pearson's r), indicating how they were calculated

Our web collection on statistics for biologists contains articles on many of the points above.

Software and code

Policy information about availability of computer code

Data collection	BD FACSDiva software (BD FACSymphony or BD LSRFortessa)<br>LAS-X software (LEICA, SP8 upright)<br>Fusion FX software (Fusion FX Vilber Lourmat)<br>Tecan software (Infinite M200 pro Tecan)
Data analysis	FlowJo software (TreeStar Inc.)<br>ImageJ 2.0 software (Fiji Software)<br>Fusion FX software<br>Tecan software<br>GraphPad Prism 7.0 software

For manuscripts utilizing custom algorithms or software that are central to the research but not yet described in published literature, software must be made available to editors and reviewers. We strongly encourage code deposition in a community repository (e.g. GitHub). See the Nature Research guidelines for submitting code & software for further information.

Data

Policy information about availability of data

All manuscripts must include a data availability statement. This statement should provide the following information, where applicable:

- Accession codes, unique identifiers, or web links for publicly available datasets
- A list of figures that have associated raw data
- A description of any restrictions on data availability

All the data generated or analyzed during this study are included in this published article and its supplementary information or available from the authors upon

reasonable request.

Field-specific reporting

Please select the one below that is the best fit for your research. If you are not sure, read the appropriate sections before making your selection.

- [x] Life sciences
- [ ] Behavioural & social sciences
- [ ] Ecological, evolutionary & environmental sciences

For a reference copy of the document with all sections, see nature.com/documents/nr-reporting-summary-flat.pdf

Life sciences study design

All studies must disclose on these points even when the disclosure is negative.

Sample size	Sample sizes were based on previous experience with similar experimental systems (Romao et al., J Cell Biol 2013).
Data exclusions	No data were excluded.
Replication	All data contain replicates and the number of replicates are stated in the figure legends..
Randomization	Samples were randomly allocated to experimental groups.
Blinding	Data were evaluated by two investigators that were blinded to experimental group allocations.

Reporting for specific materials, systems and methods

We require information from authors about some types of materials, experimental systems and methods used in many studies. Here, indicate whether each material, system or method listed is relevant to your study. If you are not sure if a list item applies to your research, read the appropriate section before selecting a response.

Materials & experimental systems

n/a	Involved in the study	
☐	☒	Antibodies
☐	☒	Eukaryotic cell lines
☒	☐	Palaeontology and archaeology
☒	☐	Animals and other organisms
☐	☒	Human research participants
☒	☐	Clinical data
☒	☐	Dual use research of concern

Methods

n/a	Involved in the study	
☒	☐	ChIP-seq
☐	☒	Flow cytometry
☒	☐	MRI-based neuroimaging

Antibodies

Antibodies used	All antibodies are listed in a table in the methods.
Validation	All are commercial antibodies and their validation can be found in the respective manufacturers' documentation.

Eukaryotic cell lines

Policy information about cell lines

Cell line source(s)	ATCC
Authentication	Authentication by the commercial source.
Mycoplasma contamination	Mycoplasma tests were performed monthly from cell culture supernatants.
Commonly misidentified lines (See ICLAC register)	None.

Human research participants

Policy information about studies involving human research participants

Population characteristics	Healthy blood donors.
Recruitment	Blood Donation Center Zurich or laboratory members.
Ethics oversight	Cantonal Ethics Committee of Zurich, Switzerland (protocols no. KEK-StV-Nr.19/08 and 2019-00837)

Note that full information on the approval of the study protocol must also be provided in the manuscript.

Flow Cytometry

Plots

Confirm that:

- [x] The axis labels state the marker and fluorochrome used (e.g. CD4-FITC).
- [x] The axis scales are clearly visible. Include numbers along axes only for bottom left plot of group (a 'group' is an analysis of identical markers).
- [x] All plots are contour plots with outliers or pseudocolor plots.
- [x] A numerical value for number of cells or percentage (with statistics) is provided.

Methodology

Sample preparation	Human monocyte-derived macrophages were analyzed after in vitro differentiation.
Instrument	BD FACSymphony or BD LSRFortessa
Software	BD FACSDiva and FlowJo
Cell population abundance	Thousands of cells were evaluated.
Gating strategy	FSC/SSC, single cell and live cell gates. Surface markers were assessed on CD14 positive myeloid cells.

- [x] Tick this box to confirm that a figure exemplifying the gating strategy is provided in the Supplementary Information.
