# Supplementary figures and images for: Oxidation inhibits autophagy protein deconjugation from phagosomes to sustain MHC class II restricted antigen presentation
Source: Nat Commun. 2021 Mar 8;12:1508. doi: 10.1038/s41467-021-21829-6 (PMC7940406; doi:10.1038/s41467-021-21829-6)

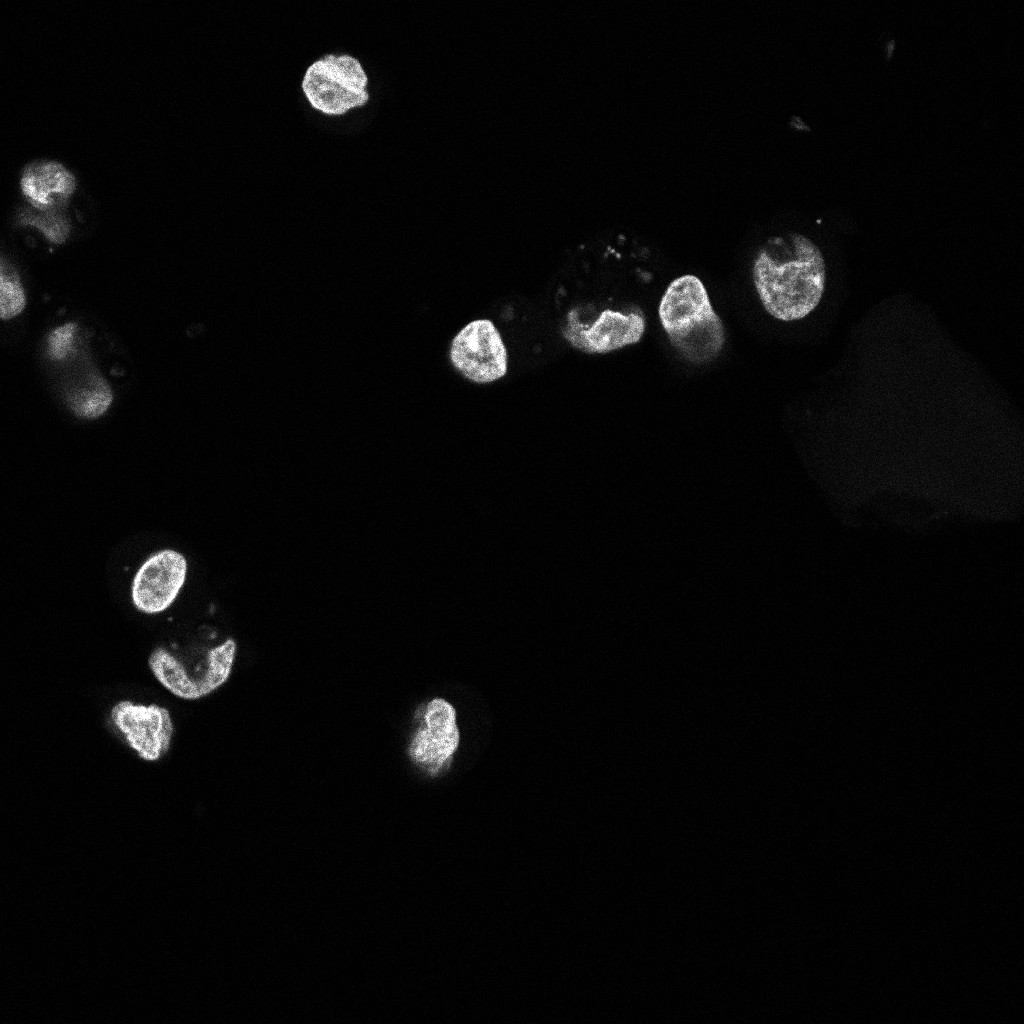

Supplement: Supplementary file 3 — Source Data [file 41467_2021_21829_MOESM3_ESM.zip › source data 110221/Fig1A.tif]

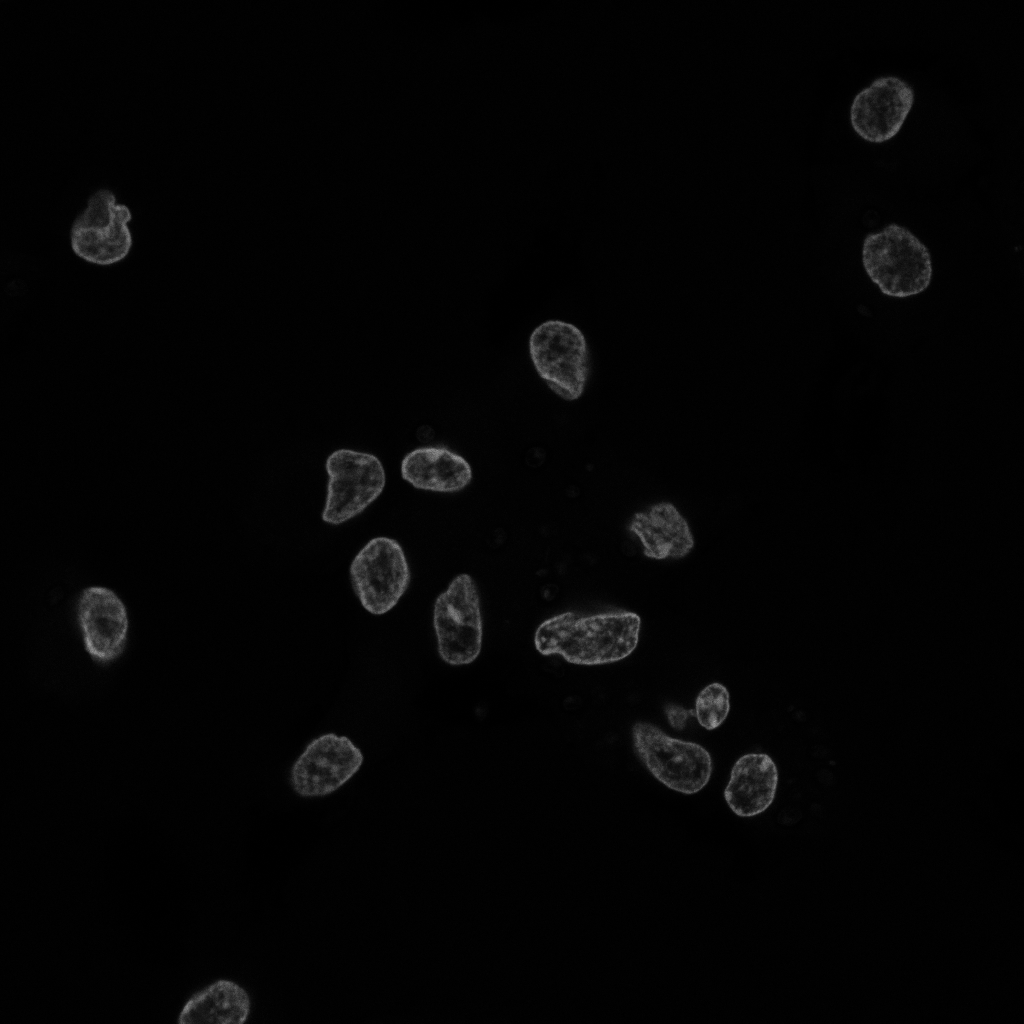

Supplement: Supplementary file 3 — Source Data [file 41467_2021_21829_MOESM3_ESM.zip › source data 110221/Fig1B.tif]

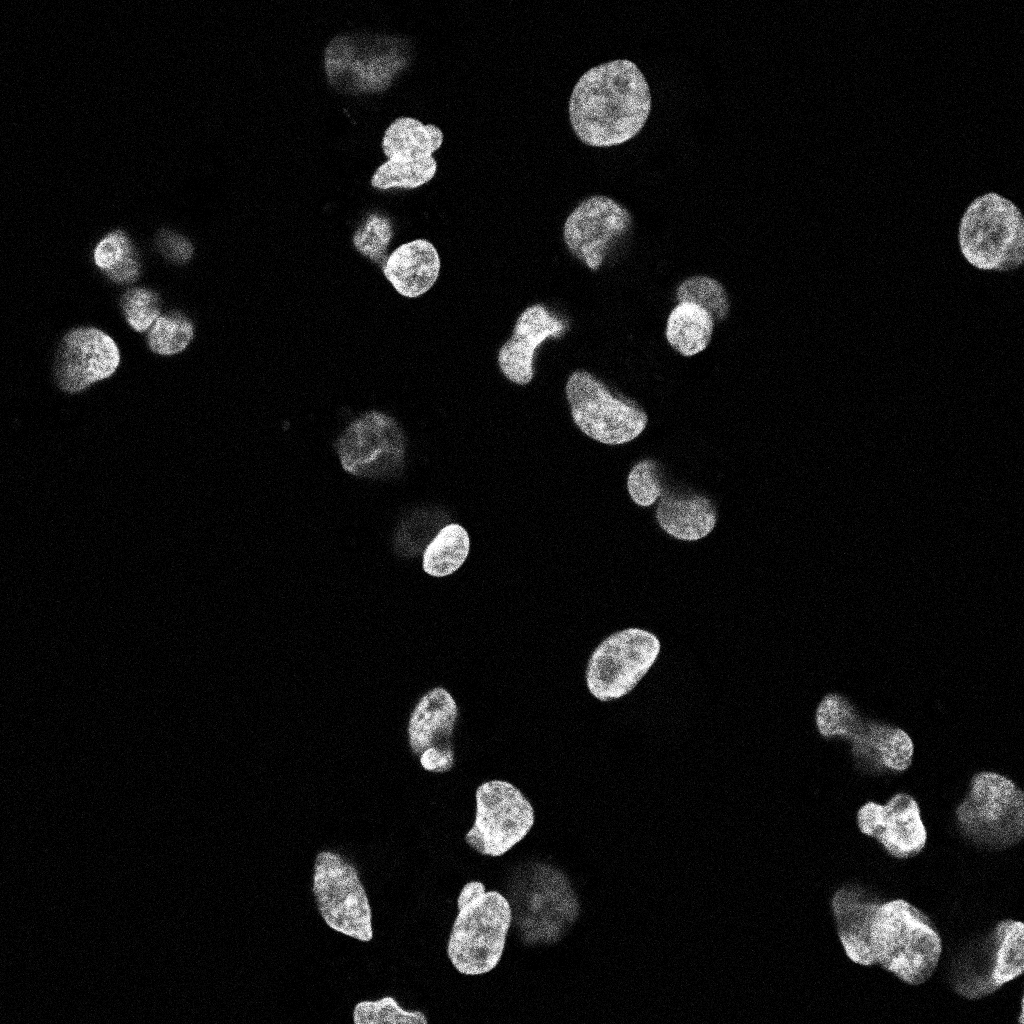

Supplement: Supplementary file 3 — Source Data [file 41467_2021_21829_MOESM3_ESM.zip › source data 110221/Fig1C.tif]

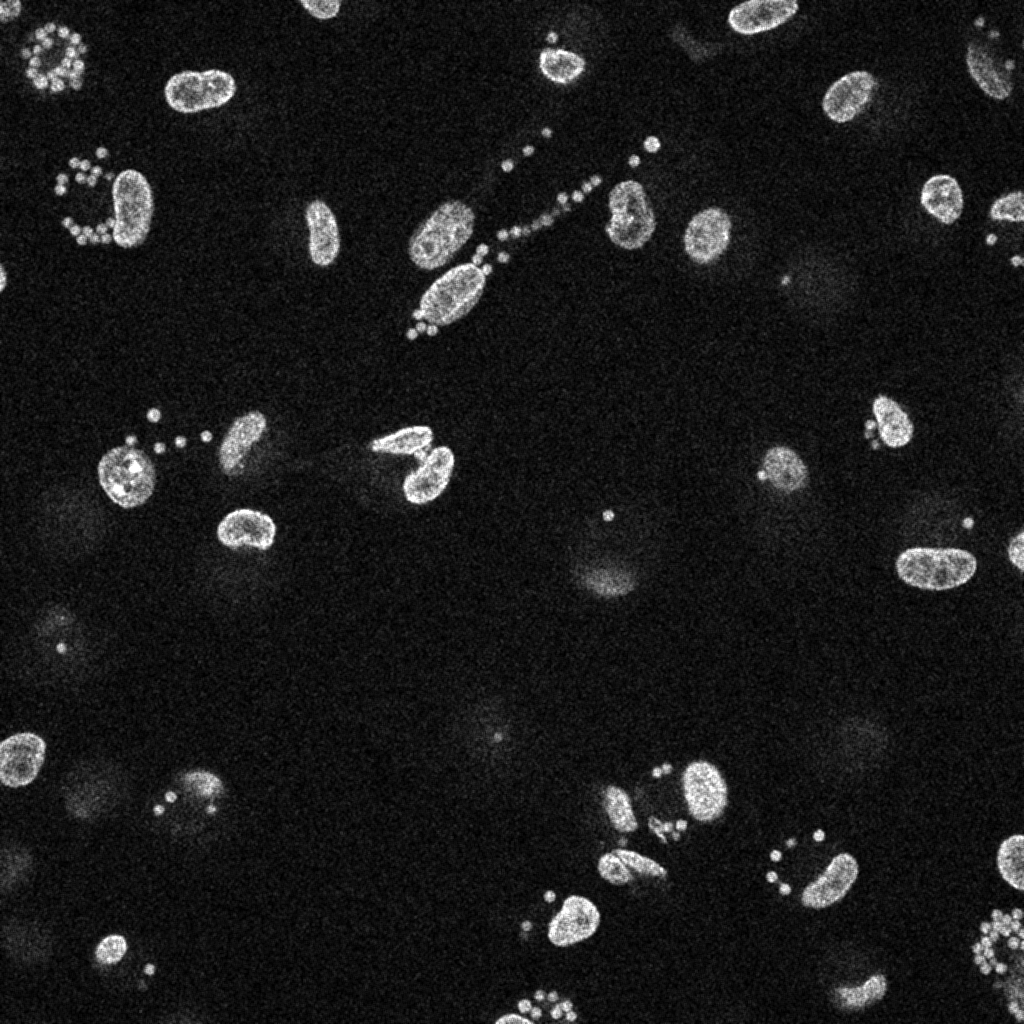

Supplement: Supplementary file 3 — Source Data [file 41467_2021_21829_MOESM3_ESM.zip › source data 110221/Fig1D beads.tif]

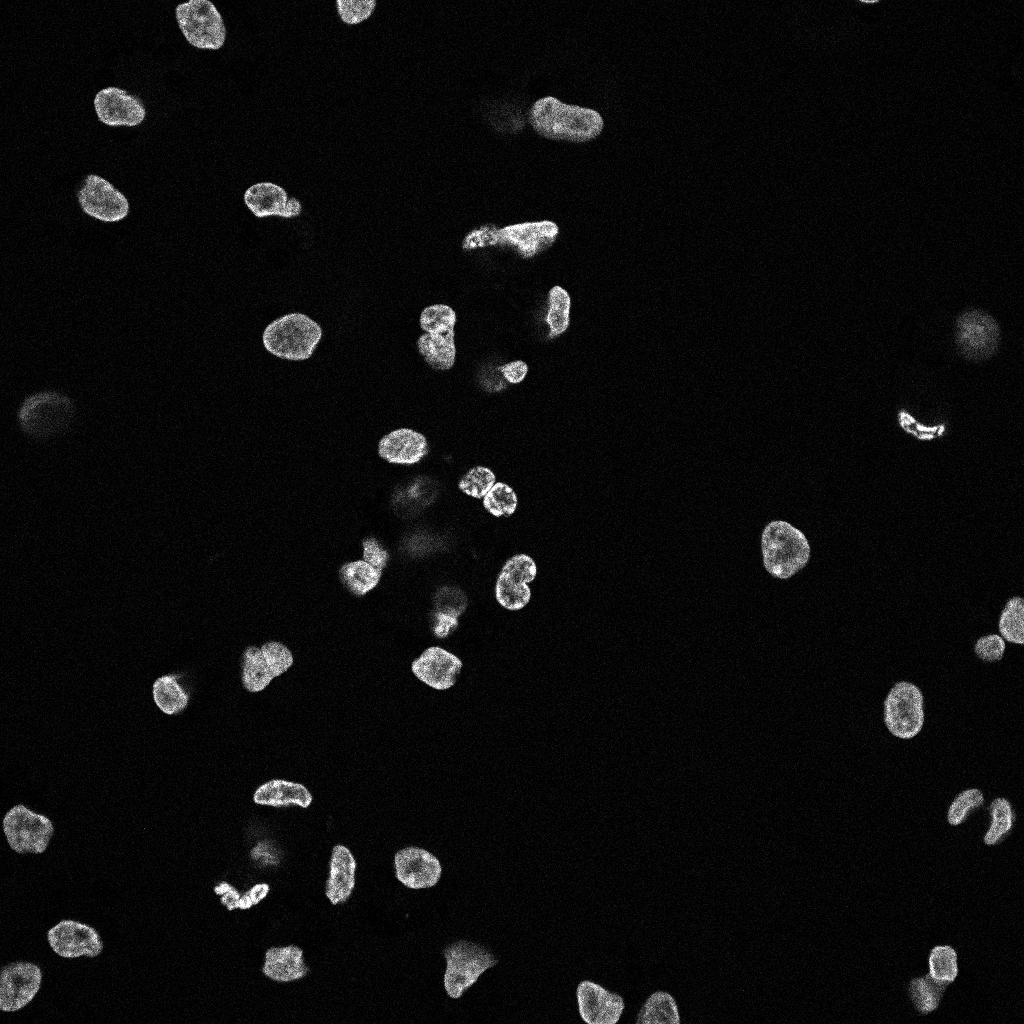

Supplement: Supplementary file 3 — Source Data [file 41467_2021_21829_MOESM3_ESM.zip › source data 110221/Fig1D zym.tif]

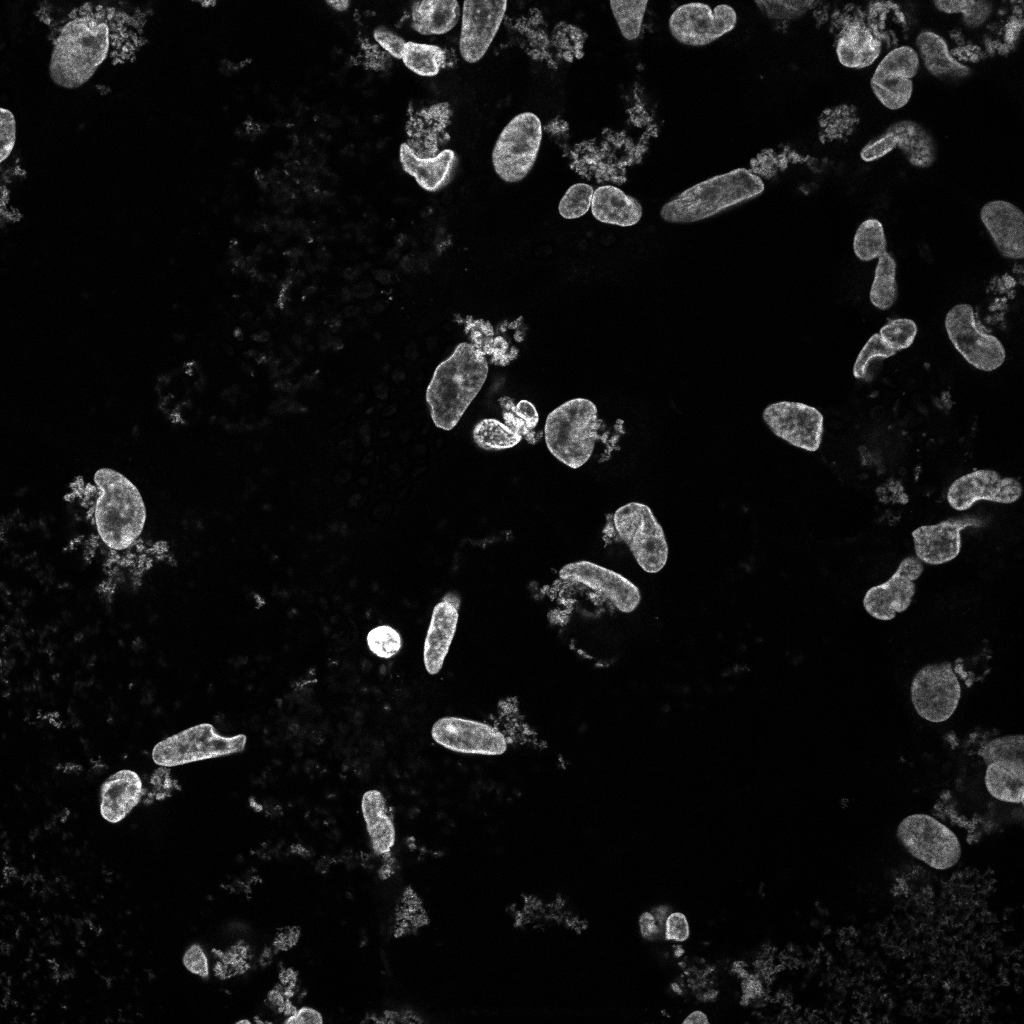

Supplement: Supplementary file 3 — Source Data [file 41467_2021_21829_MOESM3_ESM.zip › source data 110221/Fig1E.tif]

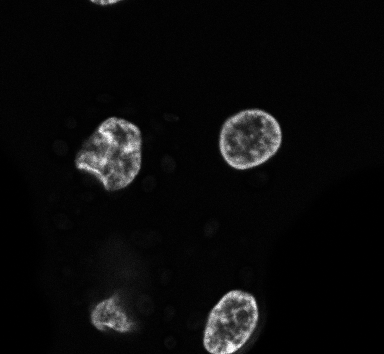

Supplement: Supplementary file 3 — Source Data [file 41467_2021_21829_MOESM3_ESM.zip › source data 110221/Fig1F .tif]

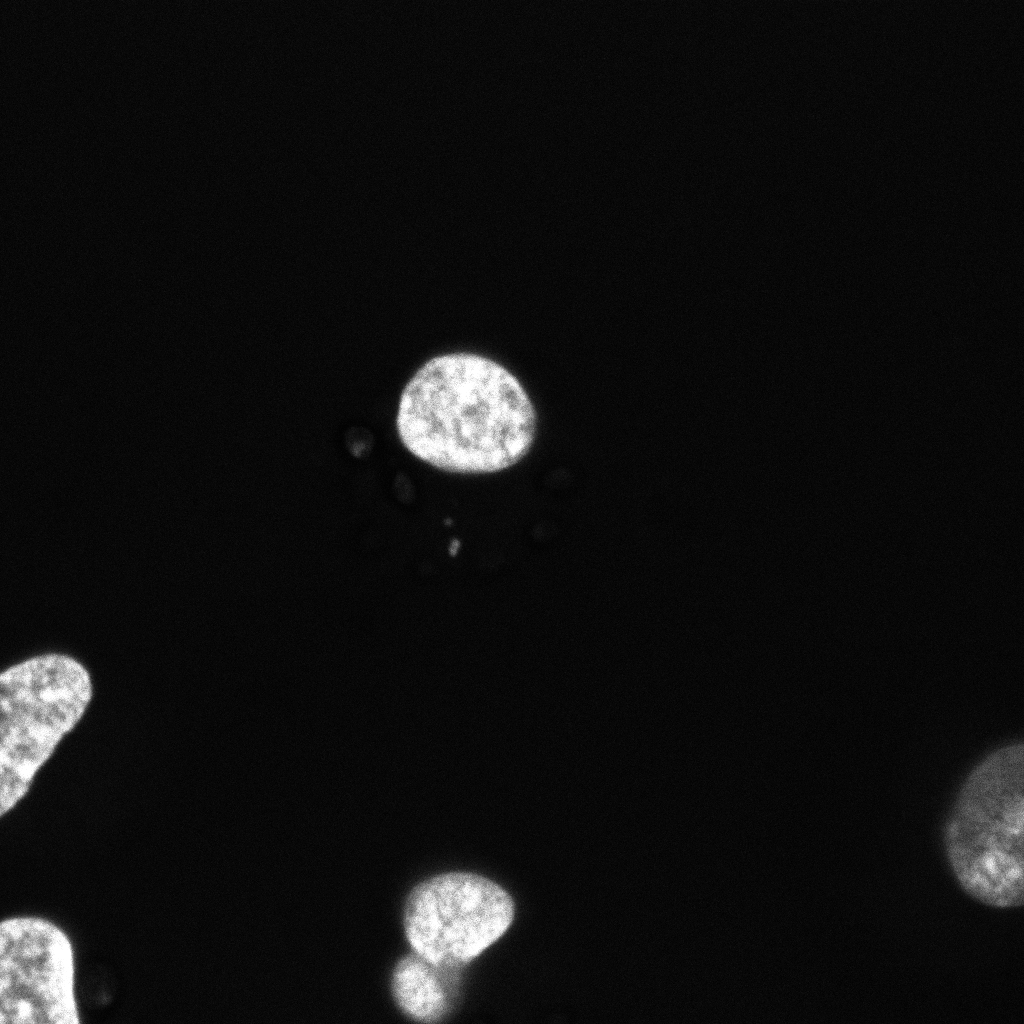

Supplement: Supplementary file 3 — Source Data [file 41467_2021_21829_MOESM3_ESM.zip › source data 110221/Fig2A.tif]

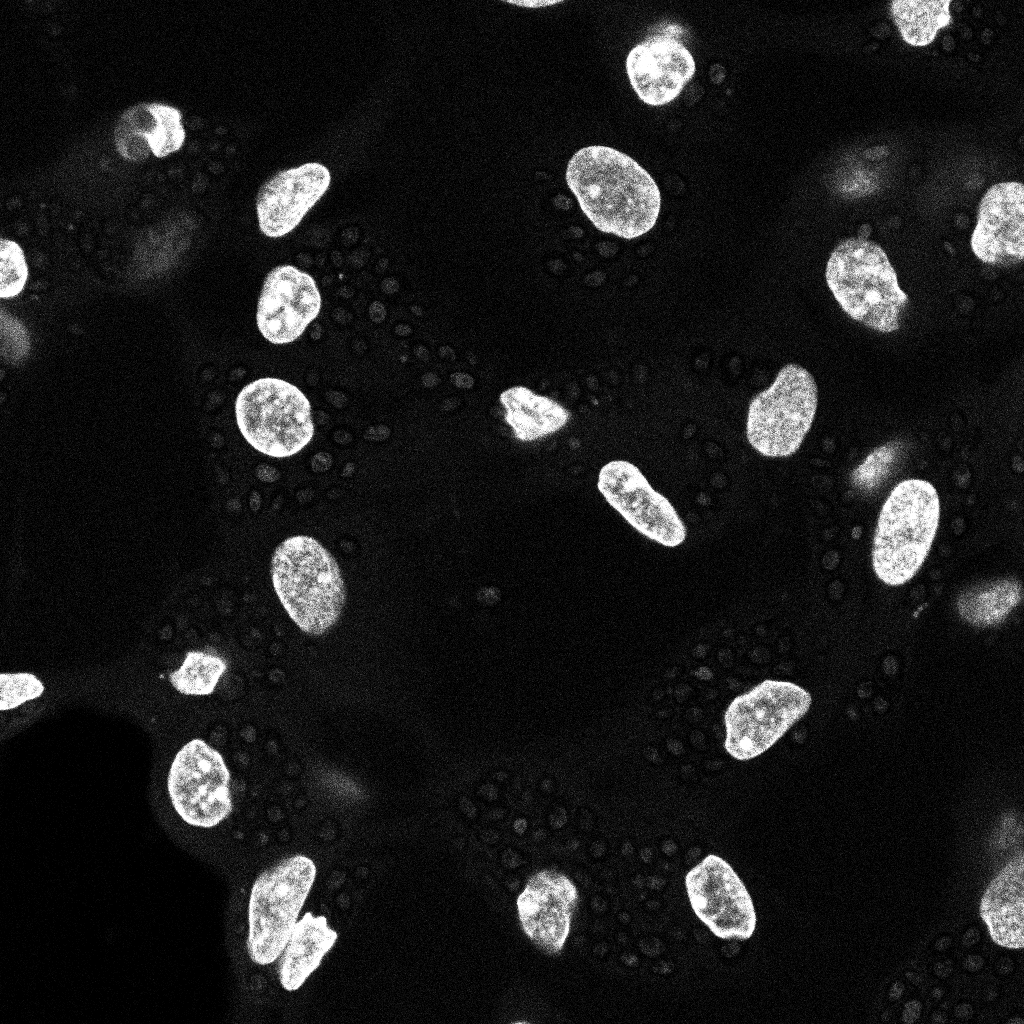

Supplement: Supplementary file 3 — Source Data [file 41467_2021_21829_MOESM3_ESM.zip › source data 110221/Fig3C Apocyanin.tif]

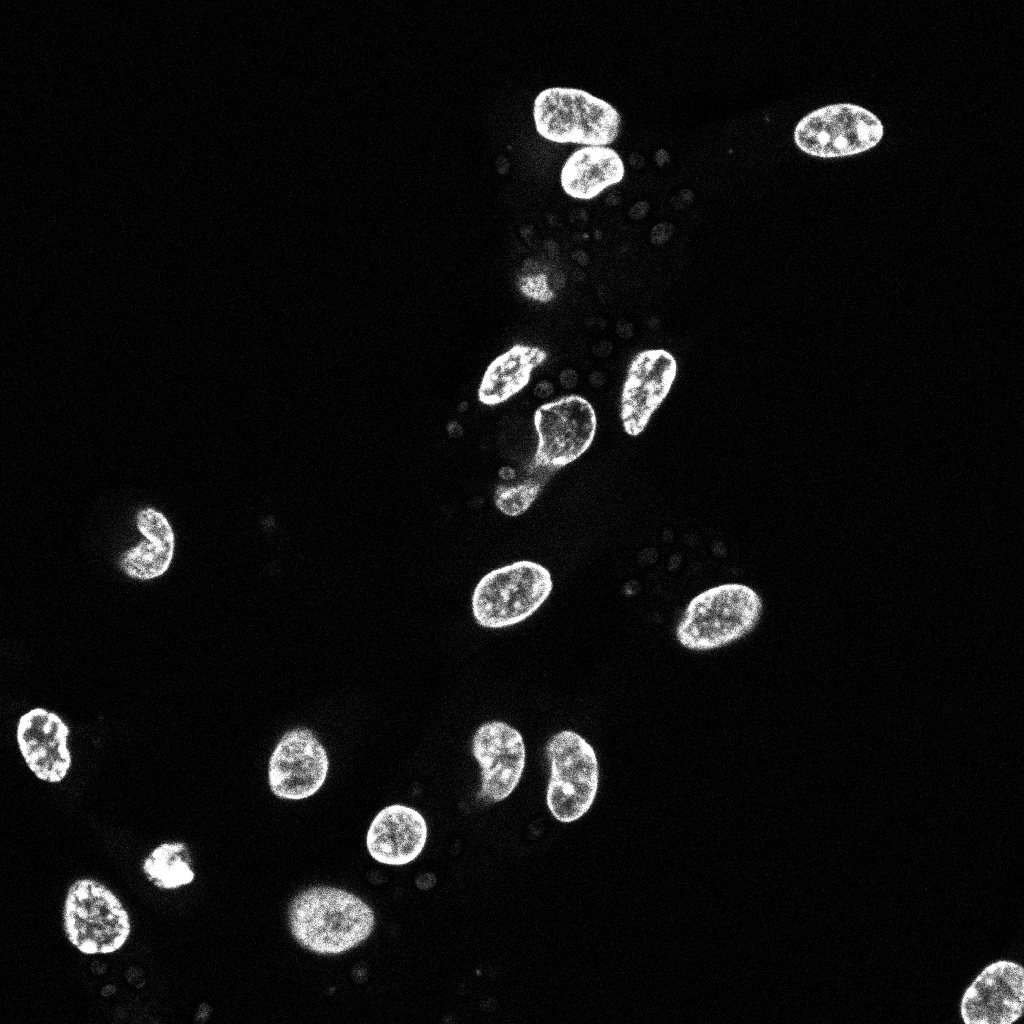

Supplement: Supplementary file 3 — Source Data [file 41467_2021_21829_MOESM3_ESM.zip › source data 110221/Fig3C DMSO.tif]

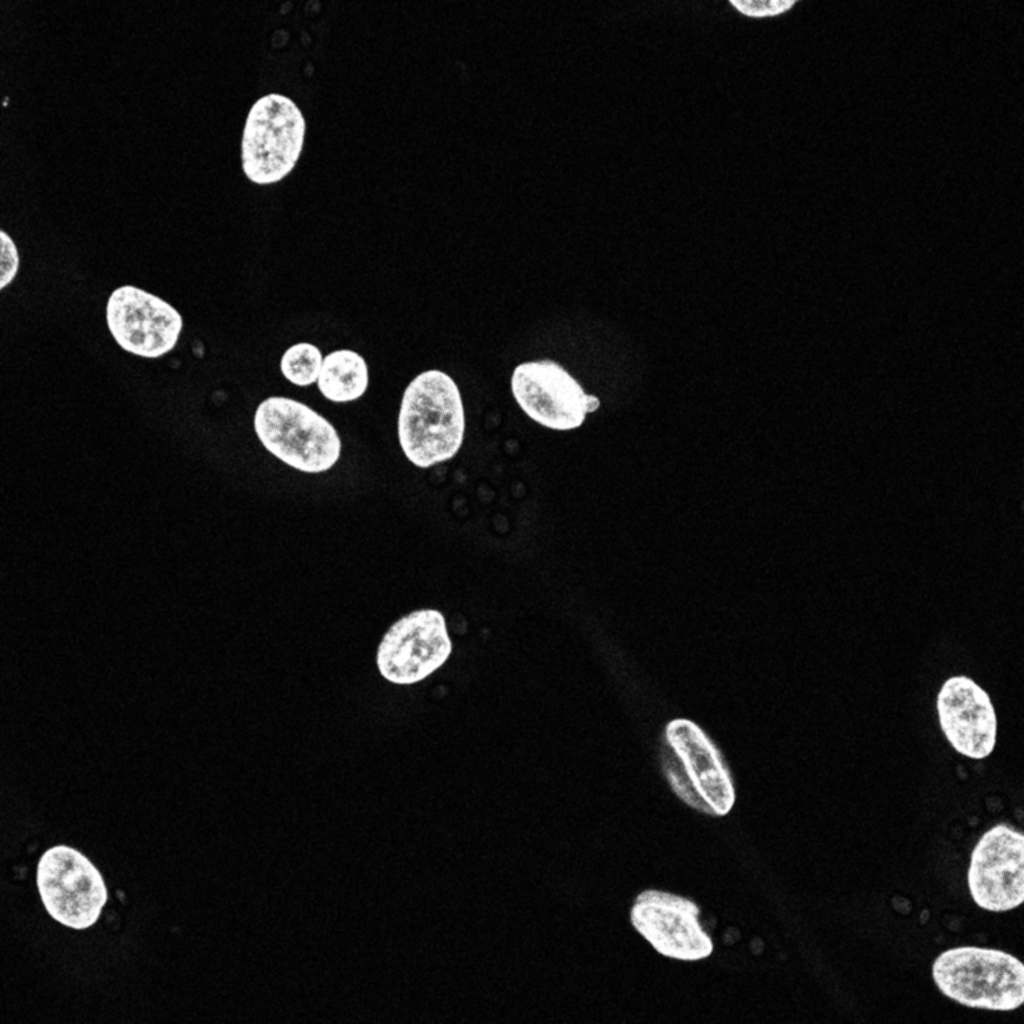

Supplement: Supplementary file 3 — Source Data [file 41467_2021_21829_MOESM3_ESM.zip › source data 110221/Fig3C DPI.tif]

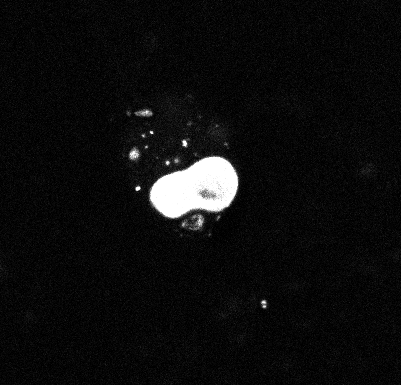

Supplement: Supplementary file 3 — Source Data [file 41467_2021_21829_MOESM3_ESM.zip › source data 110221/Fig3G ATG4B wt.tif]

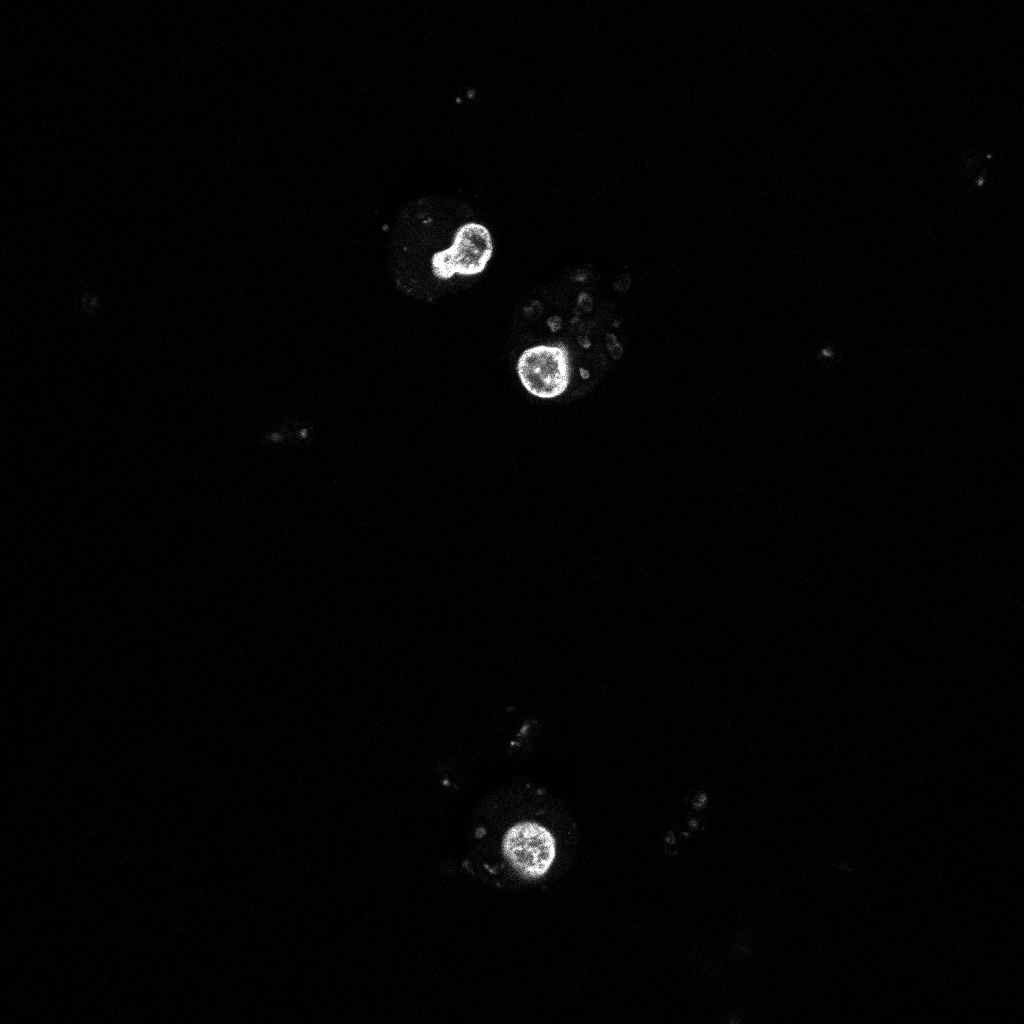

Supplement: Supplementary file 3 — Source Data [file 41467_2021_21829_MOESM3_ESM.zip › source data 110221/Fig3G ATG4BC78S.tif]

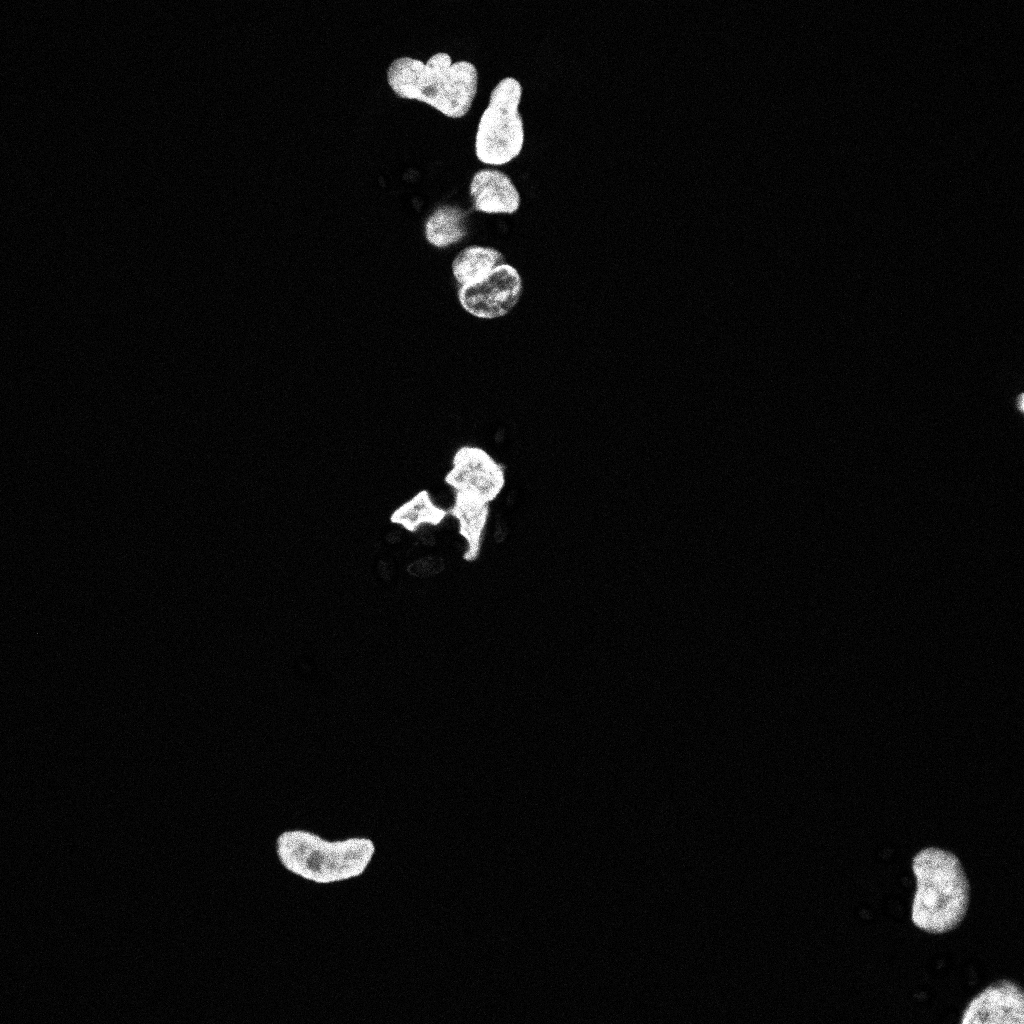

Supplement: Supplementary file 3 — Source Data [file 41467_2021_21829_MOESM3_ESM.zip › source data 110221/Fig3H.tif]

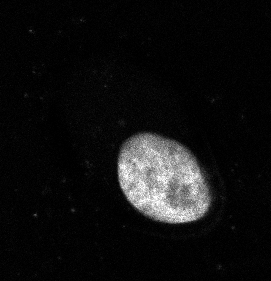

Supplement: Supplementary file 3 — Source Data [file 41467_2021_21829_MOESM3_ESM.zip › source data 110221/Fig4B ATG4C78S us.tif]

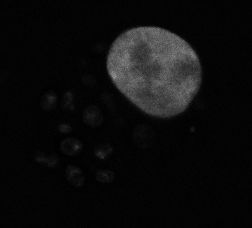

Supplement: Supplementary file 3 — Source Data [file 41467_2021_21829_MOESM3_ESM.zip › source data 110221/Fig4B ATG4C78S Zym.tif]

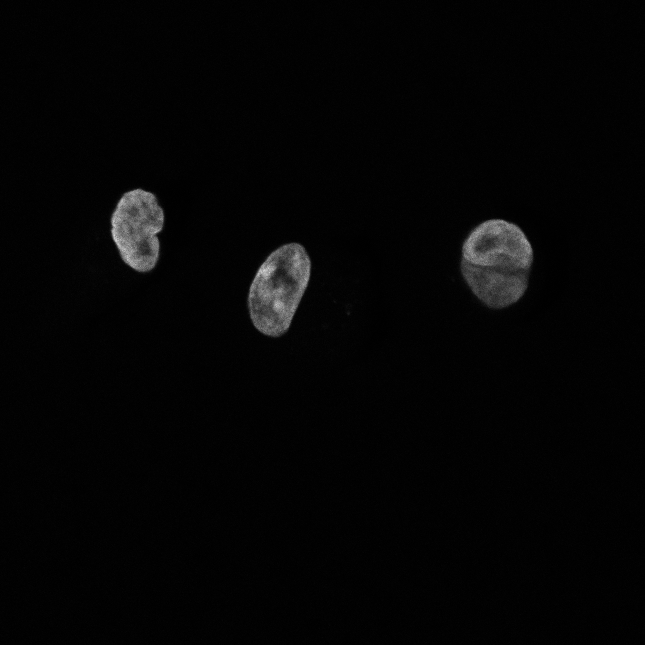

Supplement: Supplementary file 3 — Source Data [file 41467_2021_21829_MOESM3_ESM.zip › source data 110221/Fig4B ATG4Cwt US.tif]

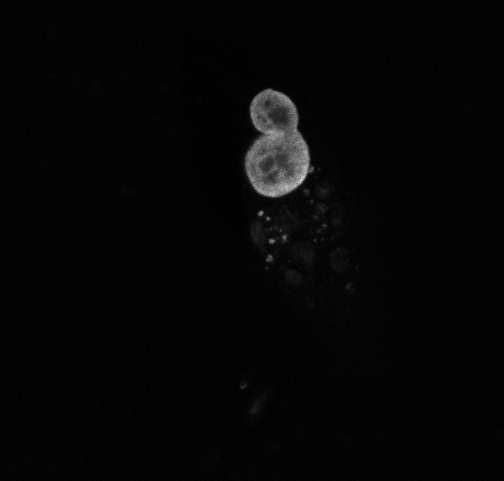

Supplement: Supplementary file 3 — Source Data [file 41467_2021_21829_MOESM3_ESM.zip › source data 110221/Fig4B ATG4CwtZym.tif]

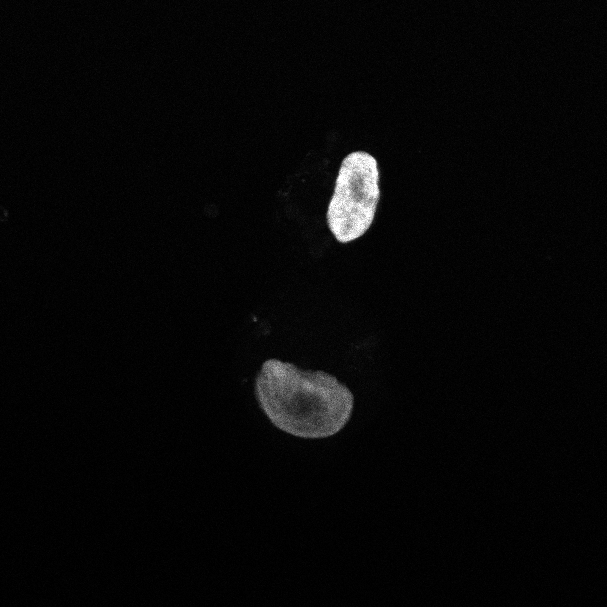

Supplement: Supplementary file 3 — Source Data [file 41467_2021_21829_MOESM3_ESM.zip › source data 110221/Fig5B ATG4B78S.tif]

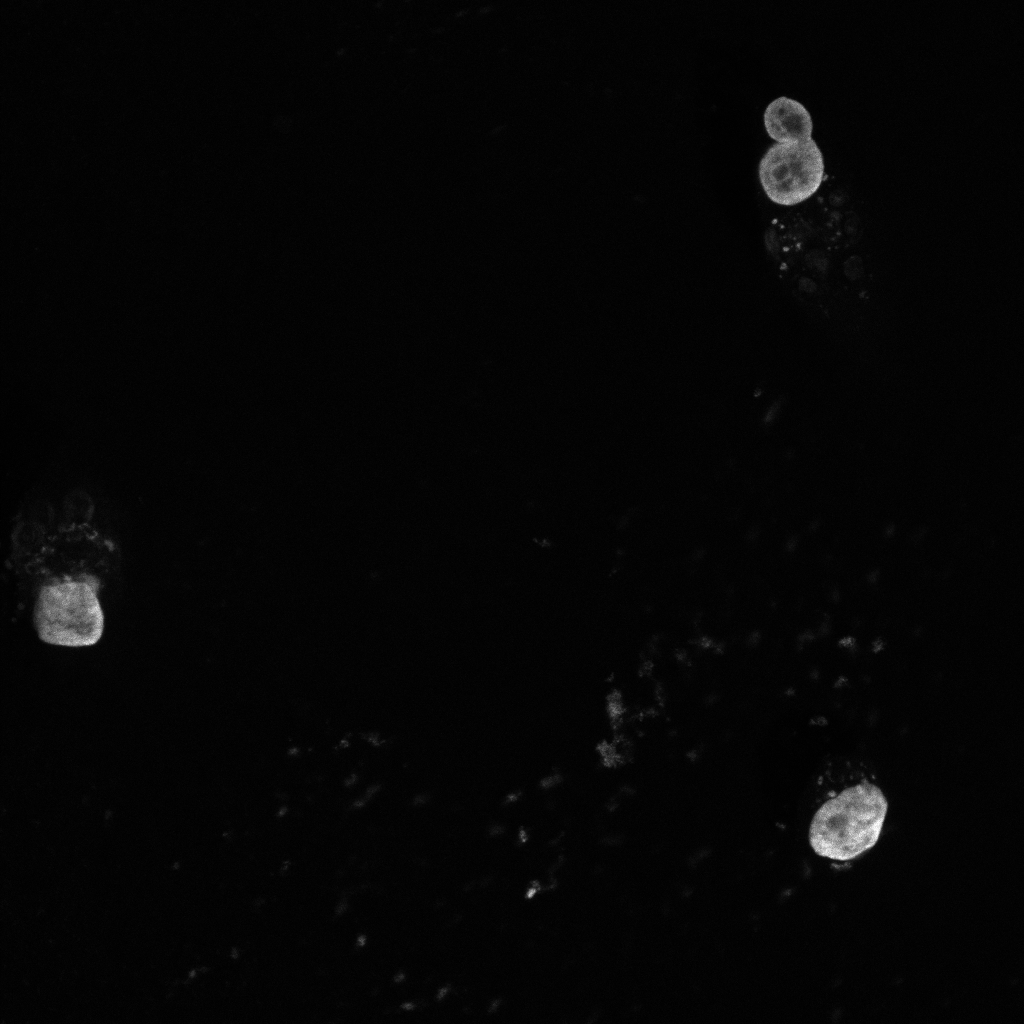

Supplement: Supplementary file 3 — Source Data [file 41467_2021_21829_MOESM3_ESM.zip › source data 110221/Fig5B ATG4BWT.tif]

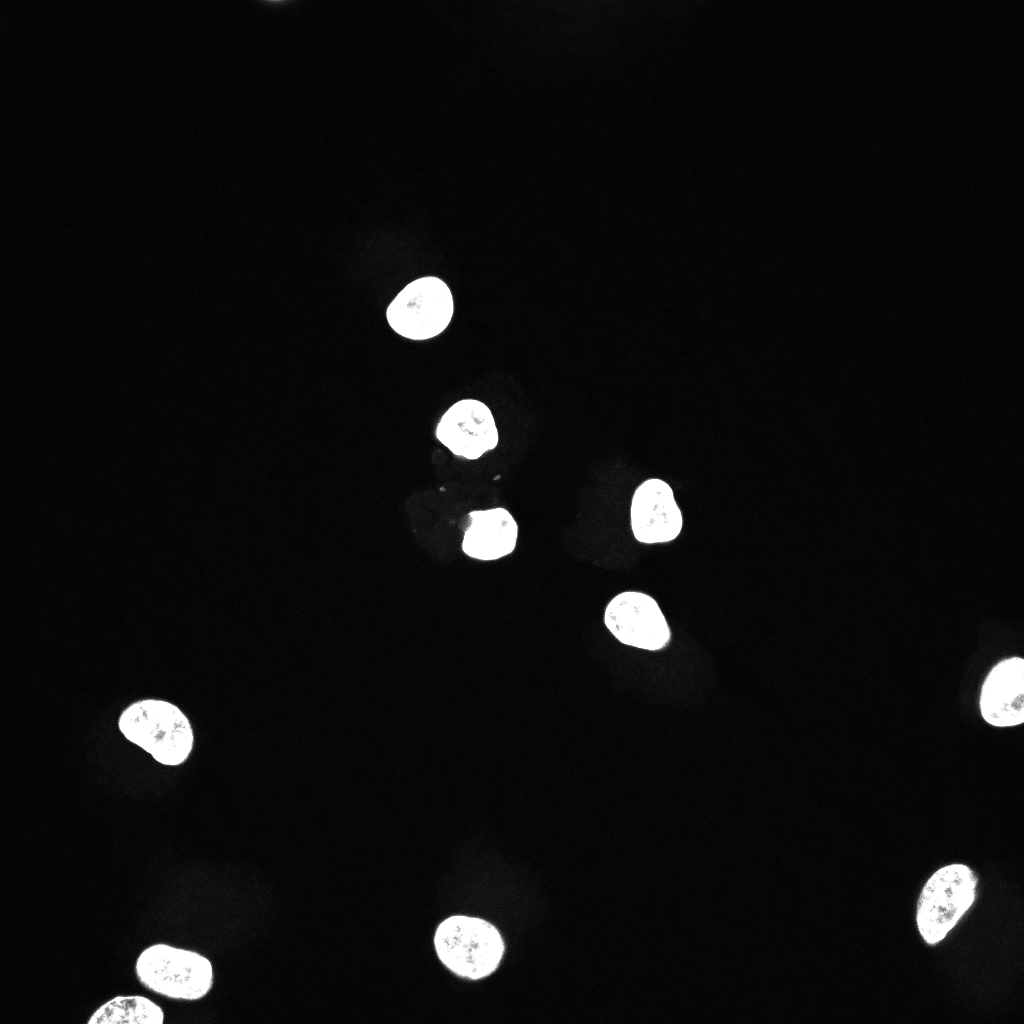

Supplement: Supplementary file 3 — Source Data [file 41467_2021_21829_MOESM3_ESM.zip › source data 110221/Fig5B Flag.tif]

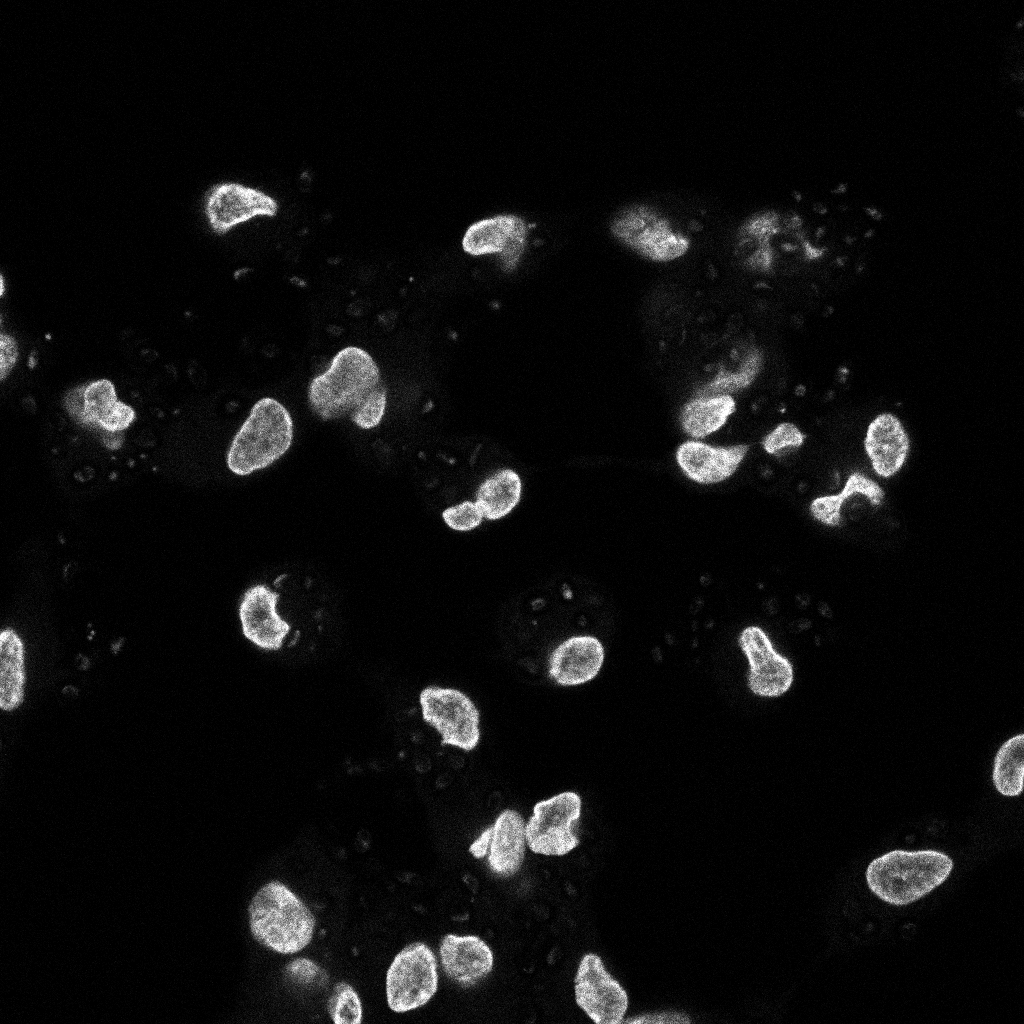

Supplement: Supplementary file 3 — Source Data [file 41467_2021_21829_MOESM3_ESM.zip › source data 110221/SupFig1A.tif]

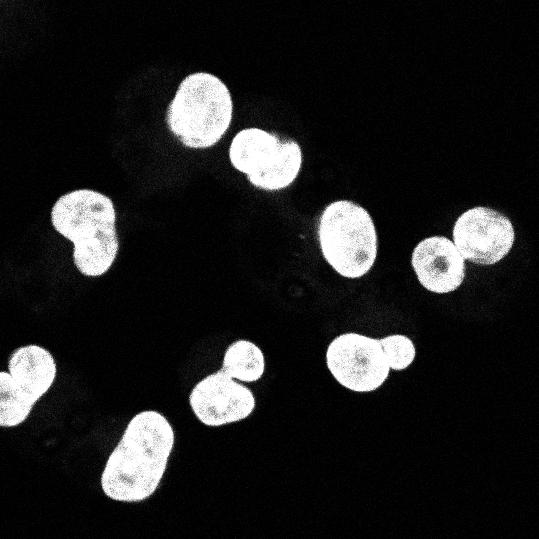

Supplement: Supplementary file 3 — Source Data [file 41467_2021_21829_MOESM3_ESM.zip › source data 110221/SupFig1C UT.tif]

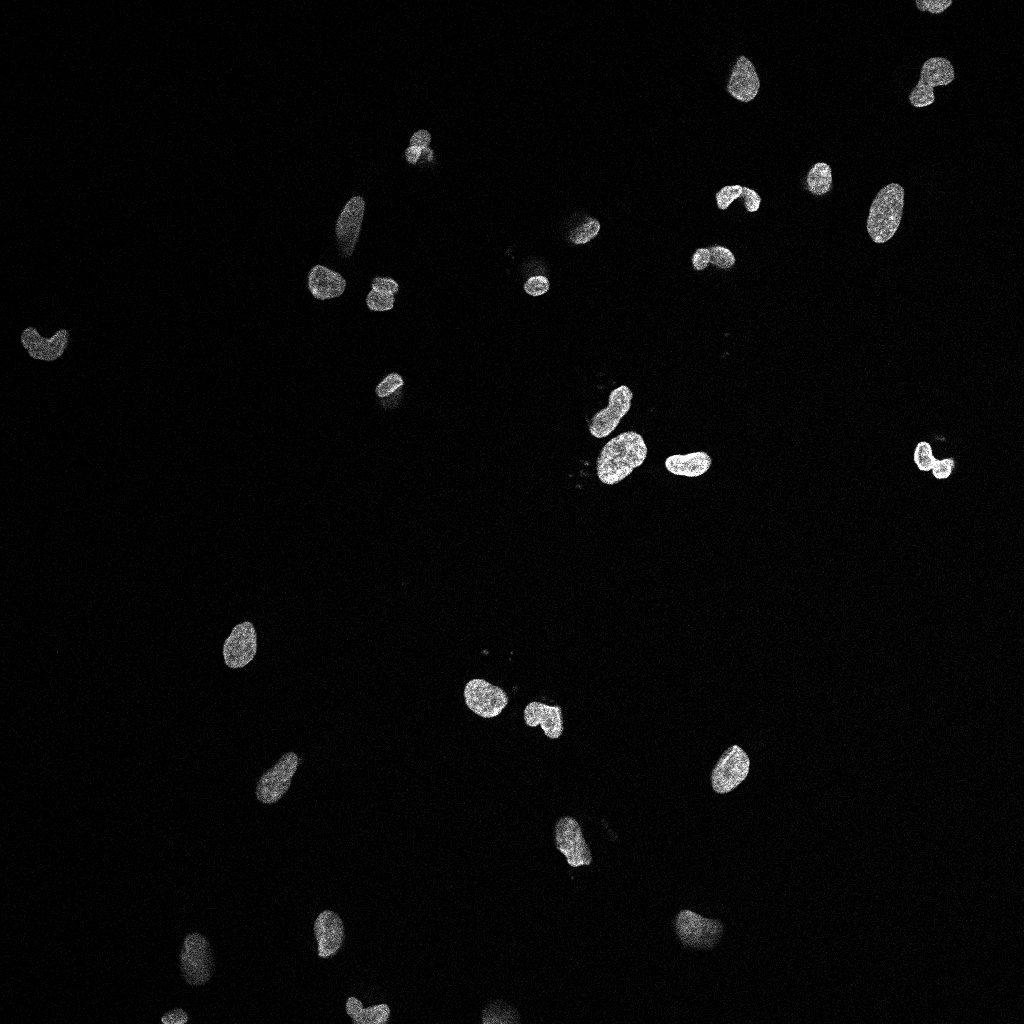

Supplement: Supplementary file 3 — Source Data [file 41467_2021_21829_MOESM3_ESM.zip › source data 110221/SupFig2A Atg12 zym.tif]

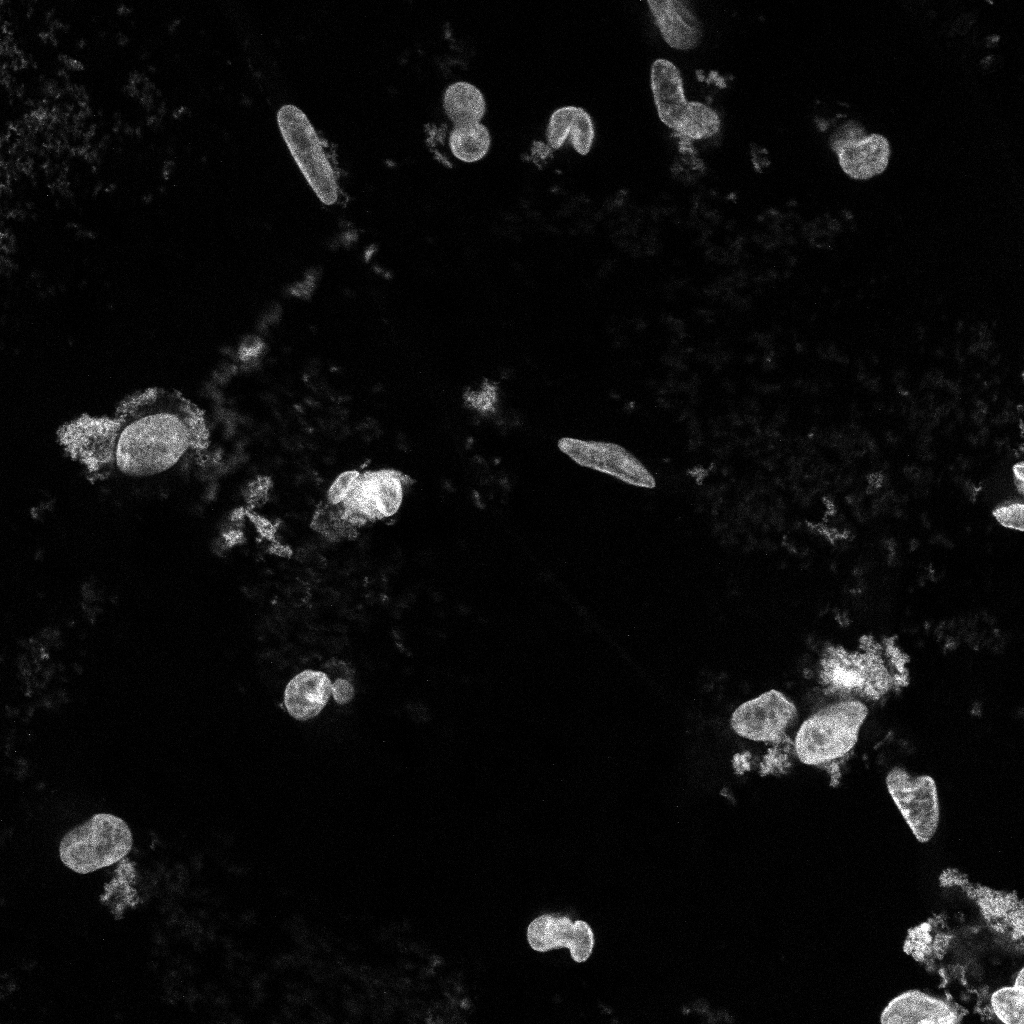

Supplement: Supplementary file 3 — Source Data [file 41467_2021_21829_MOESM3_ESM.zip › source data 110221/SupFig2C GFP-LC3 ATG12.tif]

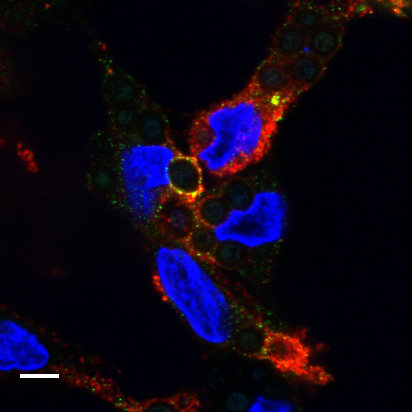

Supplement: Supplementary file 3 — Source Data [file 41467_2021_21829_MOESM3_ESM.zip › source data 110221/SupFig3B.tif]
